# Prognostic value of body adipose tissue parameters in cancer patients treated with immune checkpoint inhibitors

**DOI:** 10.3389/fimmu.2025.1557726

**Published:** 2025-02-12

**Authors:** Yan Li, Yean Yu, Kun Lv, Rongjuan Ge, Xie Xie

**Affiliations:** ^1^ Department of Traditional Chinese Medicine, Wuhan Third Hospital, Tongren Hospital of Wuhan University, Wuhan, China; ^2^ Department of Nephrology, Wuhan Third Hospital, Tongren Hospital of Wuhan University, Wuhan, China

**Keywords:** body adipose tissue, visceral adipose tissue, subcutaneous adipose tissue, immune checkpoint inhibitors, cancer

## Abstract

**Objective:**

This study aims to explore the relationship between body adipose tissue characteristics and clinical outcomes in cancer patients receiving immune checkpoint inhibitor (ICI) therapy.

**Methods:**

We conducted an extensive literature search across three major online databases—Embase, PubMed, and the Cochrane Library—to identify studies examining the link between body adipose tissue and treatment outcomes in cancer patients undergoing ICI therapy, from the inception of each database until February 20, 2024. The quality of the included studies was evaluated using the Newcastle-Ottawa Scale. The primary outcomes analyzed were hazard ratios (HRs) for overall survival (OS) and progression-free survival (PFS), as well as odds ratios (ORs) for disease control rate (DCR). Pooled estimates and 95% confidence intervals (CIs) were calculated.

**Results:**

A total of 23 studies were included, encompassing 2741 cancer patients. The analysis revealed that patients with higher levels of visceral adipose tissue (VAT) exhibited significantly improved OS (HR: 0.72, 95% CI: 0.59–0.89, *p* < 0.001) and PFS (HR: 0.80, 95% CI: 0.67–0.96, *p* = 0.015), along with a higher DCR (OR: 1.81, 95% CI: 1.26–2.60, *p* = 0.001), compared to those with lower VAT levels. Additionally, increased subcutaneous adipose tissue (SAT) levels were associated with significantly better OS (HR: 0.69, 95% CI: 0.58–0.82, *p* < 0.001) and PFS (HR: 0.82, 95% CI: 0.68–1.00, *p* = 0.049), and a higher DCR (OR: 1.99, 95% CI: 1.15–3.44, *p* = 0.014). Elevated total adipose tissue (TAT) levels were also linked to longer OS (HR: 0.73, 95% CI: 0.55–0.97, *p* = 0.028). However, a higher visceral-to-subcutaneous adipose tissue ratio (VSR) was associated with a shorter OS (HR: 1.43, 95% CI: 1.09–1.87, *p* = 0.010). No significant relationship was found between TAT (HR: 0.81, 95% CI: 0.54–1.23, *p* = 0.332) and VSR (HR: 1.20, 95% CI: 0.95–1.51, *p* = 0.131) with PFS in ICI-treated patients.

**Conclusion:**

This study highlights the prognostic relevance of VAT and SAT in predicting treatment response and survival outcomes in cancer patients receiving ICIs. These findings suggest that assessments of VAT and SAT should be incorporated into prognostic evaluations for this patient population.

## 1 Introduction

The phenomenon of immune evasion plays a pivotal role in the onset and progression of cancer and is acknowledged as one of its fundamental attributes (1). Immune checkpoints, encompassing both co-inhibitory and stimulatory signals, modulate the immune system and shield tumor cells from immune surveillance (1–3). In recent years, there has been rapid advancement in medical oncology with the emergence of immune checkpoint inhibitors (ICIs) or immunotherapies, such as nivolumab, pembrolizumab, and ipilimumab (4–7). The incorporation of ICIs has become integral in the management of various malignancies, offering an unparalleled survival advantage over conventional therapies like radiation therapy and chemotherapy (4–7). While chemotherapy primarily targets cancer cells to inhibit the cell cycle, ICIs consist of antibodies directed against programmed death 1 (PD-1), programmed death-ligand (PD-L1), or cytotoxic T-lymphocyte-associated protein 4 (CTLA-4), thus intercepting crucial regulatory signals that suppress immune responses within the tumor microenvironment (4–7). Consequently, ICIs mitigate immune suppression, enabling tumor-reactive T cells to initiate an antitumor response by harnessing the patient’s immune system to combat the malignancy (4–7).

Nonetheless, the response rate to ICI therapy exhibits considerable variability across different cancer types, typically falling within the range of 10% to 40%, with the majority of patients eventually experiencing disease progression despite initial response (8, 9). Additionally, adverse effects related to immune responses from ICI therapy can be severe or even fatal (10). Identifying individuals who are unlikely to respond to ICI therapy early on has emerged as a prominent area of focus in cancer treatment, aiming to avoid ineffective treatments and minimize the risk of adverse effects (11, 12). Currently, intra-tumor PD-L1 assays are often used as biomarkers to guide ICI therapy (13, 14). The predictive capacity of PD-L1 in clinical settings remains unsatisfactory due to the heterogeneous expression in tumor tissues (15). Other immune-related biomarkers utilized for companion diagnostics encompass tumor mutation burden and microsatellite instability (16–19). Nonetheless, their individual utility is limited in predicting outcomes (16, 17). In addition, establishing consistent criteria for quantifying these biomarkers remains challenging. Therefore, the identification of novel prognostic biomarkers capable of enhancing outcomes for cancer patients undergoing ICI treatment is of paramount importance.

The prognostic implications of obesity in different cancer types remain unclear and debated in terms of survival outcomes (20). Although certain earlier investigations have hinted at a potential link between body mass index (BMI) and overall survival (OS) in advanced cancer patients receiving immune checkpoint inhibitors (ICIs) (21, 22), others have indicated no significant relationship between BMI and clinical outcomes (23). BMI calculation is straightforward and convenient; nonetheless, it represents an imperfect metric that assigns equal weight to all aspects of body composition, resulting in notable diversity in muscle and fat mass among individuals with identical BMI values (24). Consequently, there is growing interest in precise body composition evaluations, including assessments of muscle and fat masses. Currently, the predictive role of body adipose tissue in the prognosis of patients treated with ICI is currently unknown.

Hence, this study aims to make a significant contribution by systematically synthesizing all available evidence, deepening our understanding of the clinical implications of body adipose tissue in predicting prognosis for cancer patients undergoing treatment with ICIs.

## 2 Methods

### 2.1 Search strategy

Commencing February 20, 2024, an electronic search was conducted across bibliographic databases, including EMBASE, PubMed, and the Cochrane Library. Specific search terms encompassed “immune checkpoint inhibitors” [Mesh], “ICIs”, “PD-1 Inhibitors”, “PD-L1 Inhibitors”, “CTLA-4 Inhibitors”, “Subcutaneous Adipose Index”, “Subcutaneous Fat Index”, “Visceral Adipose Index”, and “Intramuscular Adipose Index”, covering all fields. The search was restricted to English language human studies. For detailed search strategies, **Supplementary Material 1** is provided. Grey literature was sought on Google Scholar, and reference lists of eligible studies were manually scrutinized. Following Cochrane collaboration guidelines, search findings from both manual and electronic sources were consolidated within Covidence software for efficient data management.

### 2.2 Inclusion and exclusion criteria

We established specific inclusion criteria to guide article selection: (i) investigations involving patients diagnosed with cancer; (ii) utilization of ICIs as the therapeutic regimen; (iii) assessment of baseline body adipose tissue’s prognostic relevance (prior to ICI administration); and (iv) documentation of at least one of the following outcome measures: OS, progression-free survival (PFS), or disease control rate (DCR). DCR was defined as the percentage of patients who achieved complete response, partial response, or stable disease. OS was defined as the time from the start of nivolumab treatment to death from any cause. PFS was defined as the time from the start of nivolumab treatment to disease progression or death from any cause.

Exclusion criteria encompassed: (i) studies employing methodologies such as animal experimentation, literature reviews, case studies, or conference abstracts; (ii) the absence of hazard ratios (HRs) or odds ratio (OR) calculations for outcome evaluation based on either text or published data; and (iii) studies in which baseline body adipose tissue data are continuous variables. In cases where studies shared patient cohorts, preference was given to articles presenting comprehensive data and employing rigorous methodologies.

### 2.3 Data extraction and quality assessment

During the data extraction process, we gathered essential information, including authorship, publication year, study design, study period, study region, cancer type, treatments, sample size, age, gender, outcomes, and parameters related to body adipose tissue (such as assessment techniques, site of measurement, and threshold values). HRs, OR, and corresponding 95% confidence intervals (CIs) were primarily extracted from multivariate analyses; alternatively, they were obtained from univariate analyses or extracted from survival analysis plots using Engauge Digitizer software (25). The quality of observational studies was assessed using the Newcastle-Ottawa Scale (NOS), with studies scoring six or higher considered to be of high quality (26). Quality-related criteria, totaling nine points, were assigned to domains including patient selection, comparability of studies, and assessment of outcomes. All procedures, from literature retrieval and screening to data extraction and quality assessment, were conducted independently by three researchers, with any discrepancies resolved through consultation with the senior author.

### 2.4 Statistical methods

The statistical analysis was conducted using Stata 15.0. Visualization of the results was achieved through forest plots. Heterogeneity was assessed using Cochran's Q test and I^2^ statistics, with significant heterogeneity defined as a *p*-value of < 0.1 and an I^2^ value exceeding 50%. When significant heterogeneity was present, a random-effect model utilizing the DerSimonian-Laird method was applied; otherwise, a fixed-effect model employing the Inverse Variance method was utilized. The evaluation of publication bias was carried out using Egger's regression test (27) and Begg's test (28). To ensure the robustness of the results, sensitivity analyses were performed by systematically removing each study. Subgroup analyses were conducted based on methods of body composition analysis. Statistical significance was determined by a two-tailed *p*-value < 0.05.

## 3 Results

### 3.1 Search results and included studies

**Figure 1:**
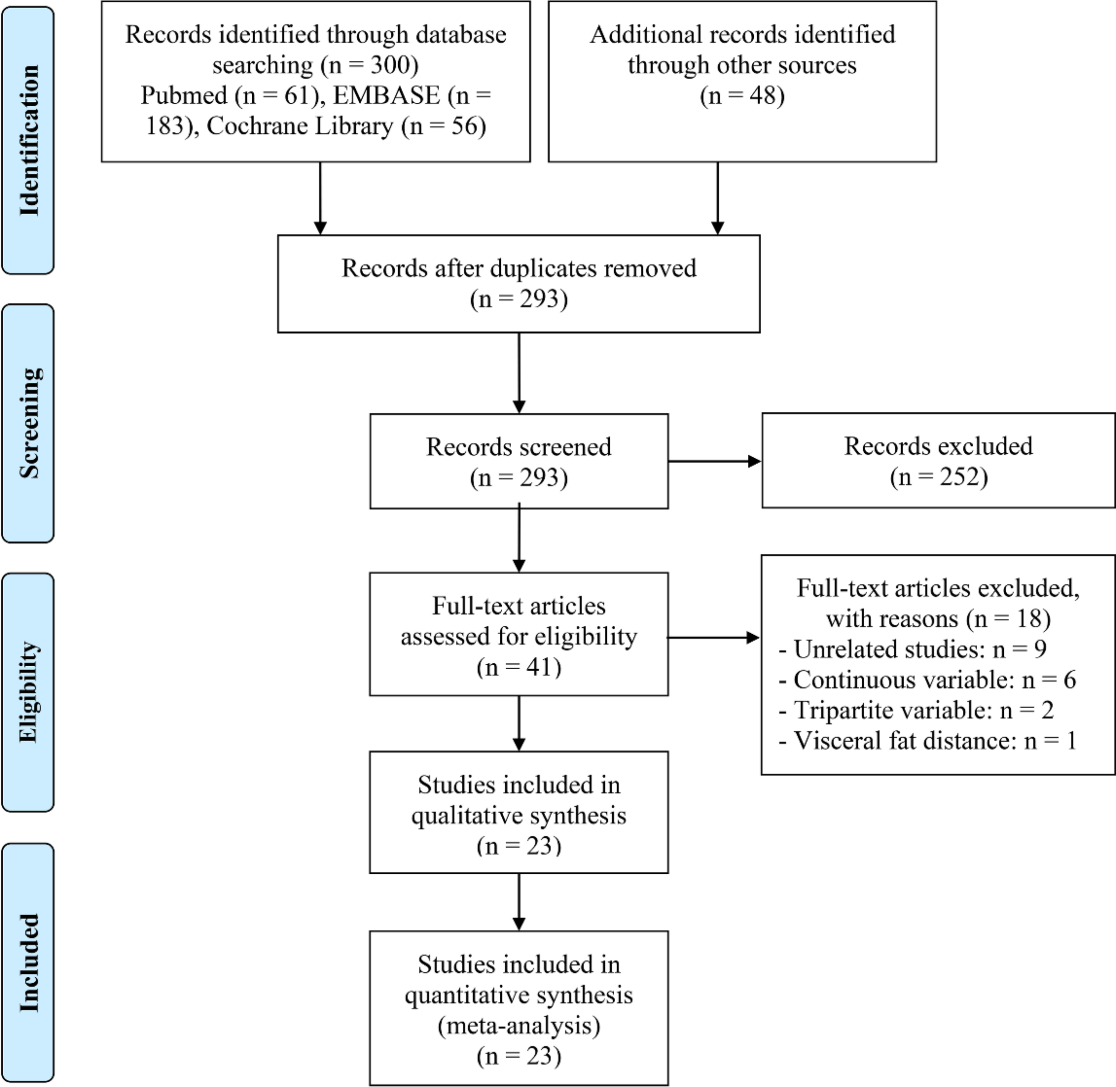
The flow diagram of identifying eligible studies.

The predetermined search strategy and manual exploration yielded 348 potentially pertinent articles. Among these, 55 duplicates were removed, and 252 were excluded based on inadequate alignment with the selection criteria outlined in their titles and abstracts. Following a comprehensive assessment of the full texts of the remaining 41 articles, 18 were excluded due to not meeting the specified criteria. Consequently, a total of 23 studies were considered eligible for inclusion (**Figure 1**) (29–51).

### 3.2 Study characteristics

**Table 1** presents the primary characteristics of the studies included in this analysis. A total of 2741 patients (68.73% male) with mean or median ages ranging from 51.4 to 72 years were included, with sample sizes varying from 44 to 623 individuals. Among these studies, five were conducted in Japan, four in China, and four in the United States. Computed tomography (CT) was employed in all studies to assess body adipose tissue at the third lumbar vertebra. All studies were retrospective, with Newcastle-Ottawa Scale (NOS) scores ranging from 6 to 8, indicating minimal risk of bias (**Table 1**).

**Table 1:** Main characteristics of the studies included.

Study	Study design	Study period	Study region	Cancer type	Treatments	Sample size	Age	Gender (male/female)	Outcome	Cut-off	Method and site	NOS
McManus et al., 2023 (44)	R	01/2015-06/2021	United States	RCC	Ipilimumab+Nivolumab	99	62 (37-81)^b^	73/26	OS, PFS	SAI: M_52.3 cm^2^/m^2^ and F_79.4 cm^2^/m^2^; VAI: M_53.3 cm^2^/m^2^ and F_32.4 cm^2^/m^2^; TAI: M_118.6 cm^2^/m^2^ and F_127.5 cm^2^/m^2^	CT, L3	7
Xiao et al., 2022 (40)	R	08/2018-10/2020	China	PLC	Nivolumab, Pembrolizumab, Sintilimab, Tislelizumab, Atezolizumab, Durvalumab, Avelumab	172	51.4 ± 11.7^c^	149/23	OS, PFS	SAI, VAI, TAI (Youden Index); VSR (M_1.33 and F_0.93)	CT, L3	7
Takenaka et al., 2022 (39)	R	03/2017-06/2020	Japan	HNSCC	Nivolumab	114	65 (23-80)^b^	85/29	OS, PFS, DCR	SAI: M_28.0 cm^2^/m^2^ and F_35.7 cm^2^/m^2^; VAI: M_10.7 cm^2^/m^2^ and F_12.8 cm^2^/m^2^; TAI: M_118.6 cm^2^/m^2^ and F_127.5 cm^2^/m^2^	CT, L3	8
Khan et al., 2023 (42)	R	03/2014-06/2019	Australia	ALC	Atezolumab, Nivolumab, Pembrolizumab	97	67.5± 10.2^c^	55/42	OS, PFS	SAI: 55.4 cm^2^/m^2^; VAI: 41.9 cm^2^/m^2^; VSR: 0.74; IMAI: 3.85 cm^2^/m^2^	CT, L3	7
Zhang et al., 2023 (11)	R	02/2018-11/2021	China	HCC	Camrelizumab, Pembrolizumab, Nivolumab, Tislelizumab, Sintilimab, Toripalimab	56	58.5 (52-70)^a^	50/6	OS, PFS	SAI: 39.27 cm^2^/m^2^	CT, L3	6
Xiong et al., 2023 (50)	R	01/2019-01/2022	China	HCC	Anti-PD-(L)1 antibodies	74	56 (35-79)^b^	63/11	OS, PFS	SAI: 23.3 cm^2^/m^2^; VAI: 30.6 cm^2^/m^2^; TAI: 33.6 cm^2^/m^2^	CT, L3	6
Wang et al., 2023 (49)	R	10/2015-04/2021	China	RCC	Anti-PD-1 antibodies	224	55 (46-63)^a^	159/65	OS, PFS	SAA: 120.95 cm^2^; VAA: 108.95 cm^2^	CT, L3	7
Uojima et al., 2023 (48)	R	01/2019-04/2022	Japan	HCC	Atezolizumab + Bevacizumab	119	72 (37-83)^b^	98/21	OS, PFS	SAI: 40.3 cm^2^/m^2^; VAI: 43.2 cm^2^/m^2^; VSR:1.04	CT, L3	7
Park et al., 2023 (45)	R	2013-2019	Korea	NSCLC	Anti-PD-(L)1 antibodies	135	66 (37-93)^b^	105/30	OS, PFS	SAI: 235.0 cm^3^/m^2^; VAI: 147.6 cm^3^/m^2^; TAI: 404.1 cm^3^/m^2^	CT, -	8
Makrakis et al., 2023 (43)	R	–	Greece	NSCLC	Nivolumab, Pembrolizumab, Atezolizumab	52	68 (39-81)^b^	43/9	OS, PFS	SAI: M_50.7 cm^2^/m^2^ and F_55.4 cm^2^/m^2^; VAI: M_45.2 cm^2^/m^2^ and F_31.2 cm^2^/m^2^; IMAI: M_9.9 cm^2^/m^2^ and F_10.5 cm^2^/m^2^	CT, L3	6
Martini et al., 2023 (34)	R	2015-2020	United States	RCC	ICIs	79	61^f^	58/21	OS, PFS, DCR	SAI: M_51.4 cm^2^/m^2^ and F_69.8 cm^2^/m^2^; VAI: M_ 35.2 cm^2^/m^2^ and F_37.4 cm^2^/m^2^; TAI: M_98.7 cm^2^/m^2^ and F_94.3 cm^2^/m^2^; IMAI: M_4.4 cm^2^/m^2^ and F_7.8 cm^2^/m^2^	CT, L3	7
Martini et al., 2023 (35)	R	2015-2020	United States	UC	Pembrolizumab, Atezolizumab	70	69.5^f^	49/21	OS, PFS, DCR	SAI: M_26.2 cm^2^/m^2^ and F_53.9 cm^2^/m^2^; VAI: M_ 26.1 cm^2^/m^2^ and F_50.9 cm^2^/m^2^; IMAI: M_7.8 cm^2^/m^2^ and F_5.8 cm^2^/m^2^	CT, L3	7
Aslan et al., 2022 (36)	R	10/2010-10/2021	Turkey	RCC	Nivolumab	52	22/30^e^	38/14	OS, PFS	SAI: 55 cm^2^/m^2^	CT, L3	7
Lee et et al., 2022 (38)	R	06/2015-05/2021	Korea	Melanoma	Pembrolizumab, Nivolumab	266	60 (51-69)^a^	135/131	OS, PFS, DCR	VAI: 25 cm^2^/m^2^	CT, L3	7
Popinat et al., 2019 (29)	R	02/2015-10/2017	France	NSCLC	Nivolumab	55	63.5 (37.8-82.4)^b^	41/14	OS	SAM: 5.69 kg/m^2^; VAM:1.32 kg/m^2^; TAM: 7 kg/m^2^	CT, -	6
Martini et al., 2020 (30)	R	2009-2017	Georgia	Solid cancer	ICIs	90	–	53/37	OS, PFS	SAI: 73 cm^2^/m^2^	CT, L3	7
Baldessari et al., 2021 (32)	R	07/2017-12/2018	Italy	NSCLC	Pembrolizumab	44	70 (42-83)^b^	26/18	PFS	VSR: -	CT, L3	7
Faron et al., 2021 (33)	R	01/2013-08/2019	Germany	Melanoma	Nivolumab, Pembrolizumab, Ipilimumab	107	62 ± 15^c^	70/37	OS	SAI: M_77.4 cm^2^/m^2^ and F_38.1 cm^2^/m^2^; VAI: M_69.3 cm^2^/m^2^ and F_30.7 cm^2^/m^2^	CT, L3	8
Decazes et al., 2023 (41)	R	06/2014-12/2018	France	Melanoma and NSCLC	Pembrolizumab, Nivolumab, Ipilimuma	623	63 (22-92)^b^	353/270	OS	SAM: 3.95 kg/m^2^; VAM: 0.91 kg/m^2^; TAM: 5.26 kg/m^2^	CT, -	8
Minami et al., 2020 (31)	R	12/2015-11/2018	Japan	NSCLC	Nivolumab, Pembrolizumab or Atezolizumab	74	37^d^	48/26	OS, PFS, DCR	VAA: 100 cm^2^; IMAC: M_-0.358 and F_-0.229; VSR: M_1.33 and F_0.93	CT, L3	7
Bolte et al., 2022 (37)	R	2015-2021	United States	NSCLC	Pembrolizumab	92	64 (36-89)^b^	48/44	OS	IMAC: -	CT, L3	7
Tanaka et al., 2023 (47)	R	10/2017-12/2022	Japan	GC	Nivolumab	47	–	39/8	OS, PFS, DCR	IMAC: -0.6	CT, L3	7
Takei et al., 2023 (46)	R	2019-2023	Japan	RCC	ICIs	60	71 (63-75)^a^	46/14	OS, PFS	SAI: M_28.7 cm^2^/m^2^ and F_60.5 cm^2^/m^2^; VAI: M_ 44.1 cm^2^/m^2^ and F_27.8 cm^2^/m^2^; TAI: M_77.3 cm^2^/m^2^ and F_89.5 cm^2^/m^2^	CT, L3	7

^a^Median with interquartile range;

^b^Median with range;

^c^Mean ± standard deviation;

^d^Age ≥70 years;

^e^Age ≥65 years;

^f^median age.

R, retrospective study; CT, computed tomography; L3, 3th lumbar vertebra; ICI, immune checkpoint inhibitors; RCC, renal cell carcinoma; NSCLC, non-small cell lung cancer; PLC, primary liver cancer; HNSCC, head and neck squamous cell carcinoma; LC, Lung cancer; HCC, hepatocellular carcinoma; UC, urothelial carcinoma; GC, gastric cancer; OS, overall survival; PFS, progression-free survival; ORR, objective response rate; DCR, disease control rate; SAI, subcutaneous adipose index; SAA, subcutaneous adipose area; SAM, subcutaneous adipose mass; VAI, visceral adipose index; VAA, visceral adipose area; VAM, visceral adipose mass; TAI, total adipose index; TAM, total adipose mass; VSR, visceral-to-subcutaneous fat tissue ratio; IMAI, intramuscular adipose index; IMAC, intramuscular adipose content.

### 3.3 Baseline visceral adipose tissue and overall survival and progression-free survival

**Figure 2:**
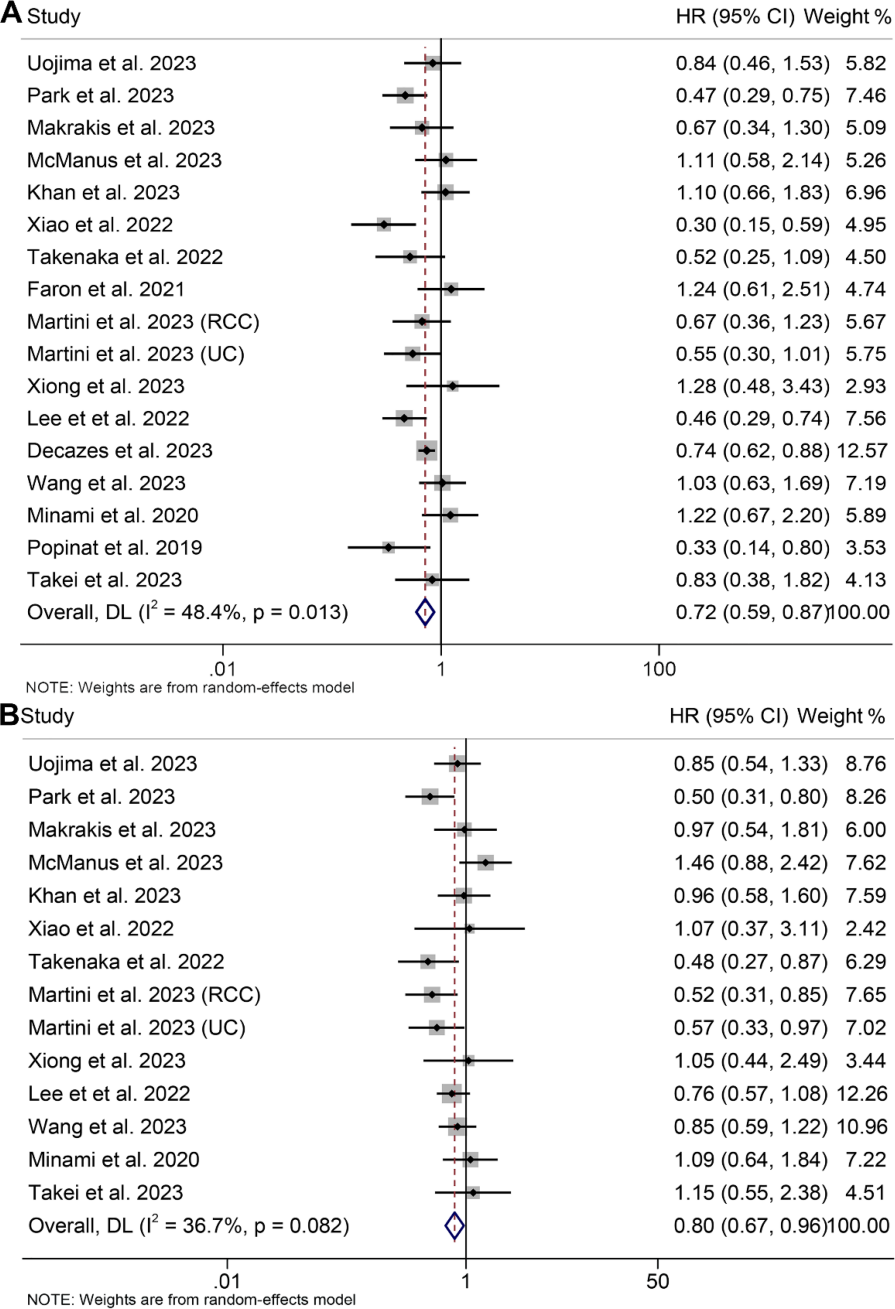
Forest plots of the relationship between visceral adipose tissue and overall survival **(A)** and progression-free survival **(B)**. HR, hazard ratio; CI, confidence interval; DL, DerSimonian and Laird.

In this analysis, we included a total of 17 studies comprising 2420 patients to investigate the effect of high and low VAT on OS or PFS in cancer patients treated with ICIs. Our findings showed that patients with high VAT had significantly longer OS (HR: 0.72, 95% CI: 0.59–0.89, *p* < 0.001, **Figure 2A**) and PFS (HR: 0.80, 95% CI: 0.67–0.96, *p* = 0.015, **Figure 2B**) than patients with low VAT. The Cochran Q test and I^2^ statistics (OS: I^2^ = 48.4%, *p* = 0.013; PFS: I^2^ = 36.7%, p = 0.082) showed that there was significant heterogeneity across studies. Consequently, a random-effects model was employed.

**Figure 3:**
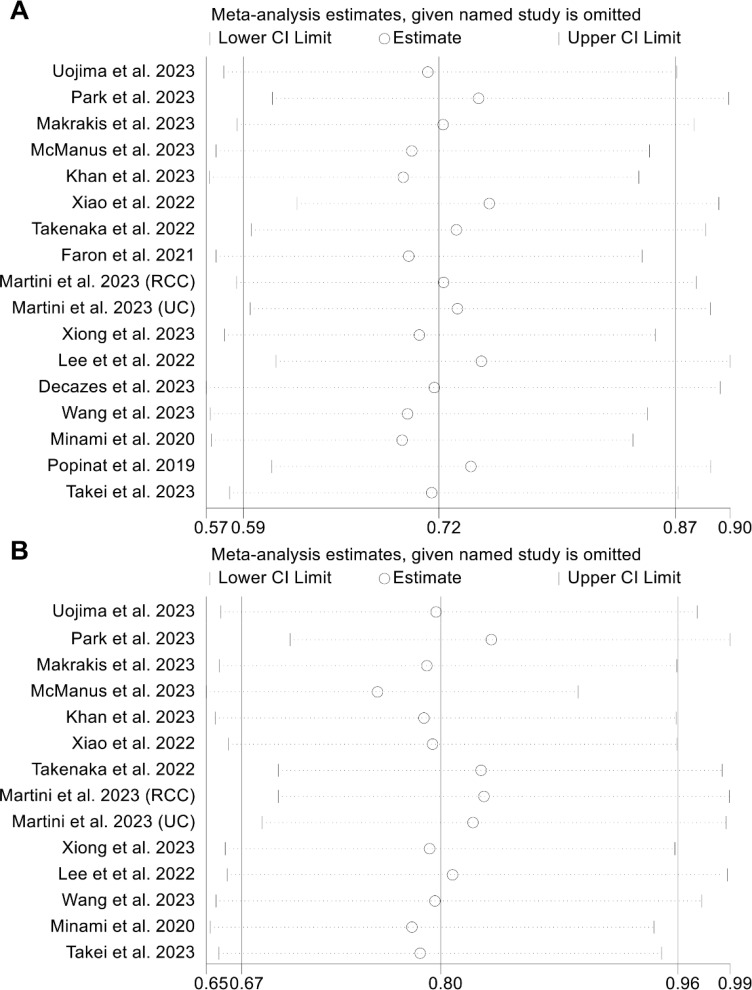
Sensitivity analysis of the association between visceral adipose tissue and overall survival **(A)** and progression-free survival **(B)**. HR, hazard ratio; CI, confidence interval.

Examination of potential publication bias through funnel plots, Begg’s test, and Egger’s test did not yield significant findings concerning OS (Egger’s test: *p* = 0.881, Begg’s test: *p* = 0.967, **Supplementary Figure S1A**) and PFS (Egger’s test: *p* = 0.546, Begg’s test: *p* = 0.511, **Supplementary Figure S1B**). Our sensitivity analysis, involving the systematic exclusion of each study in turn, consistently demonstrated the sustained stability and robustness of the pooled HRs for both OS and PFS (**Figures 3A, B**). Subgroup analyses confirmed that high visceral fat index was significantly associated with longer OS and PFS, while visceral fat area and mass were not (**Table 2**).

**Table 2:** Subgroup analysis of the association between body adipose tissue and the outcomes of cancer patients treated with immune checkpoint inhibitors.

Variable	Included studies	Test of association			Test of heterogeneity		
		HR	95%CI	*p*-value	Modal	I^2^	*p*-value
Visceral adipose tissue (OS)							
Visceral adipose index	13	0.69	0.54-0.87	***p* = 0.002**	R	45.7%	*p* = 0.036
Visceral adipose area	2	0.56	0.26-1.19	*p* = 0.128	R	68.5%	*p* = 0.075
Visceral adipose mass	2	1.10	0.76-1.61	*p* = 0.611	R	0	*p* = 0.668
Visceral adipose tissue (PFS)							
Visceral adipose index	12	0.78	0.63-0.96	***p* = 0.017**	R	41.6%	*p* = 0.064
Visceral adipose area	2	0.92	0.68-1.24	*p* = 0.588	R	0	*p* = 0.447
Subcutaneous adipose tissue (OS)							
Subcutaneous adipose index	15	0.69	0.55-0.87	***p* = 0.002**	R	43.6%	*p* = 0.036
Subcutaneous adipose mass	2	0.68	0.51-0.90	***p* = 0.007**	R	14.65%	*p* = 0.279
Subcutaneous adipose area	1	0.76	0.47-1.24	*p* = 0.271	R	–	*-*
Subcutaneous adipose tissue (PFS)							
Subcutaneous adipose index	14	0.84	0.68-1.00	***p* = 0.049**	R	46.9%	*p* = 0.027
Subcutaneous adipose area	1	0.69	0.48-1.00	***p* = 0.048**	R	–	*-*
Total adipose tissue (OS)							
Total adipose index	6	0.77	0.49-1.22	*p* = 0.266	R	53.5%	*p* = 0.056
Total adipose mass	2	0.70	0.59-0.83	***p* < 0.001**	R	0	*p* = 0.406
Total adipose tissue (PFS)							
Total adipose index	6	0.81	0.54-1.23	*p* = 0.332	R	58.5%	*p* = 0.034
Intramuscular adipose tissue (OS)							
Intramuscular adipose index	4	0.95	0.57-1.58	*p* = 0.834	R	66.7%	*p* = 0.029
Intramuscular adipose content	3	0.98	0.40-2.45	*p* = 0.973	R	79.6%	*p* = 0.007
Intramuscular adipose tissue (PFS)							
Intramuscular adipose index	4	1.00	0.62-1.62	*p* = 0.997	R	69.3%	*p* = 0.020
Intramuscular adipose content	2	0.74	0.27-2.05	*p* = 0.563	R	77.5%	*p* = 0.035

HR, hazard ratio; CL, confidence interval; OS, overall survival; PFS, progression-free survival; R, random-effect model. Bold font means p<0.05.

### 3.4 Baselinevisceral adipose tissue and immunotherapy responses

**Figure 4:**
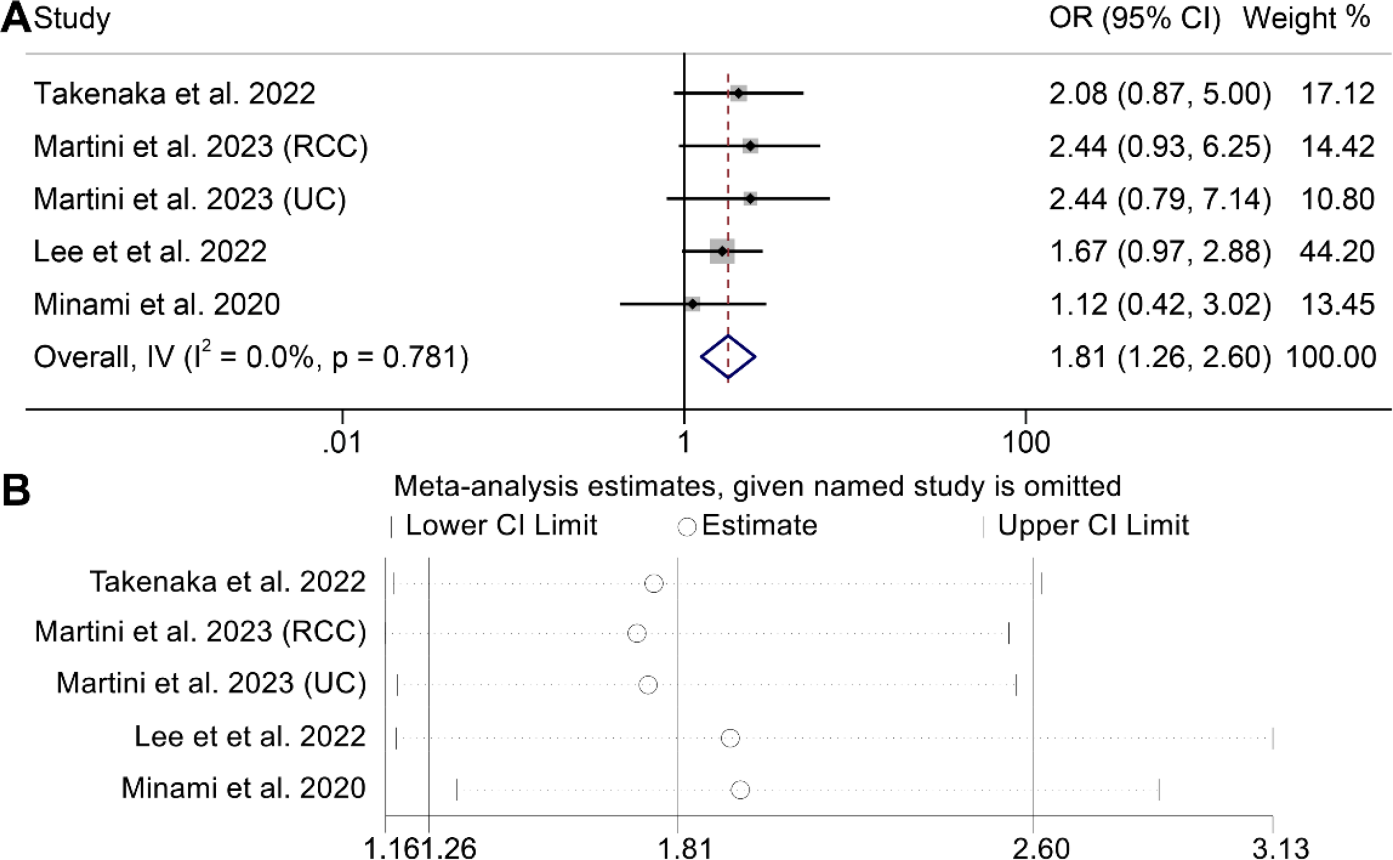
Forest plots of the relationship between visceral adipose tissue and disease control rate **(A)**. Sensitivity analysis of the association between visceral adipose tissue and disease control rate **(B)**. OR, odds ratio; CI, confidence interval; IV, Inverse Variance.

We conducted an analysis of the association between VAT and DCR in cancer patients (5 studies with 603 patients). It is noteworthy that the included studies exhibited no significant heterogeneity (I^2^ = 0, *p* = 0.781), thus warranting the application of a fixed-effects model. The results made it clear that patients with high VAT had a higher DCR (OR: 1.81, 95% CI: 1.26–2.60, *p* = 0.001, **Figure 4A**) than people with low VAT. The Egger's test (*p* = 0.643) and Begg's test (*p* = 0.806) confirmed the absence of publication bias, while sensitivity analysis revealed the stability of the results (**Figure 4B**).

### 3.5 Pre-immunotherapy subcutaneous adipose tissue and prognosis and response

**Figure 5:**
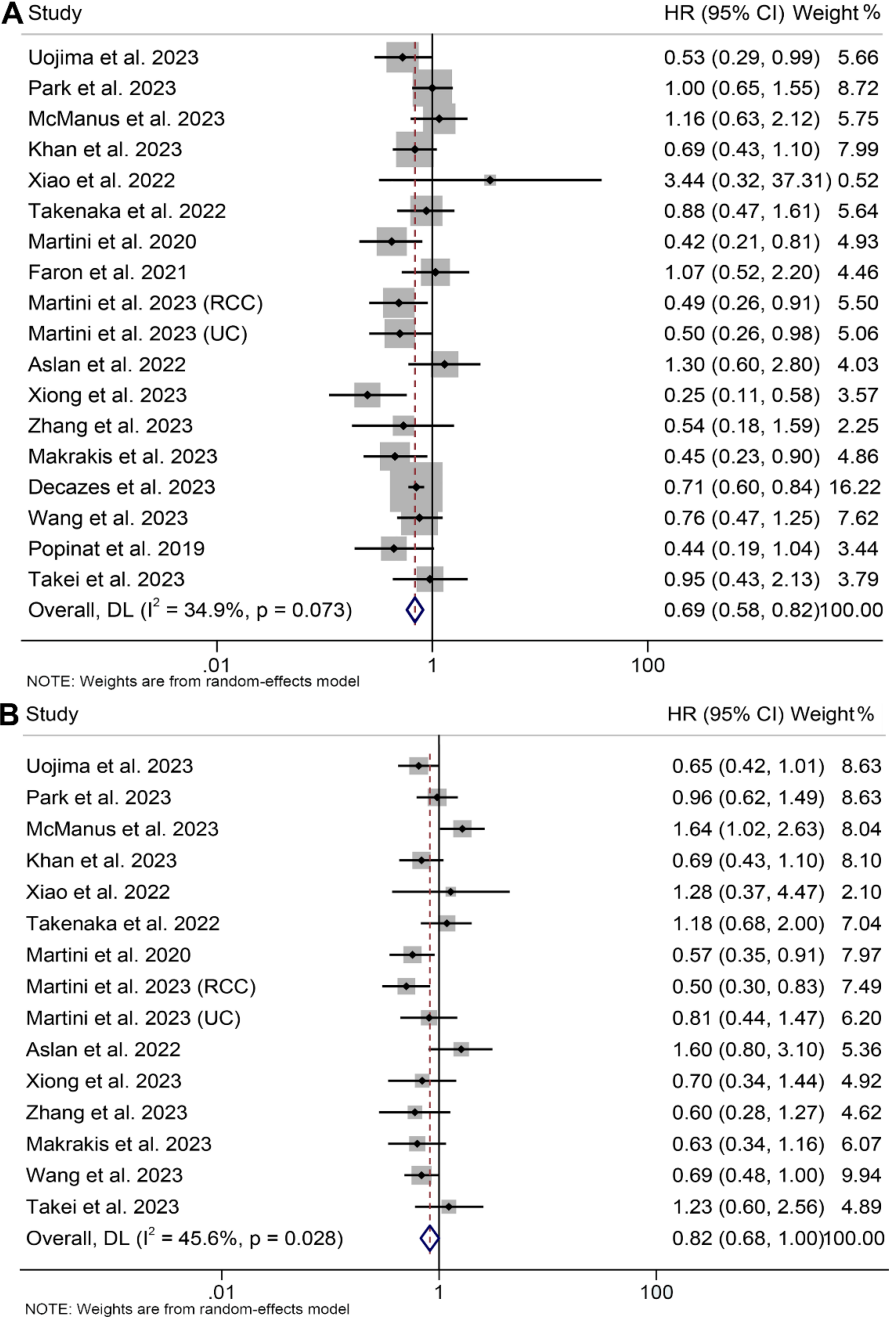
Forest plots of the relationship between subcutaneous adipose tissue and overall survival **(A)** and progression-free survival **(B)**. HR, hazard ratio; CI, confidence interval; DL, DerSimonian and Laird.

In the evaluation of the association between subcutaneous adipose tissue and survival outcomes in cancer patients, 18 cohorts comprising 2262 patients were analyzed. A random-effects model revealed that cancer patients with high SAT had a significantly improved OS (I^2^ = 34.9%, *p* = 0.073, HR: 0.69, 95% CI: 0.58–0.82, *p* < 0.001, **Figure 5A**) and PFS (I^2^ = 45.6%, *p* = 0.028, HR: 0.82, 95% CI: 0.68–1.00, *p* = 0.049, **Figure 5B**). The results of the subgroup analyses are detailed in **Table 2**.

Potential publication bias was assessed through funnel plots, Begg's test, and Egger's test, with no significant findings observed for OS (Egger's test: *p* = 0.771, Begg's test: *p* = 0.449, **Supplementary Figure S2A**) or PFS (Egger's test: *p* = 0.420, Begg's test: *p* = 0.488, **Supplementary Figure S2B**). Our sensitivity analyses, involving the systematic exclusion of each study in turn, consistently demonstrated the continued stability and robustness of the combined HRs for OS (**Supplementary Figures S3A**). However, it is worth noting that the relationship between SAT and PFS became insignificant after excluding some studies (**Supplementary Figures S3B**).

Besides, three studies involving 263 patients investigated the relationship between SAT and DCR in cancer patients undergoing ICI immunotherapy. There was no notable heterogeneity among the studies (I^2^ = 38.2%, *p* = 0.198), leading to the application of a fixed-effect model. The synthesised findings indicated that high SAT correlated with higher DCR (OR: 1.99, 95% CI: 1.15–3.44, *p* = 0.014, **Supplementary Figure S4**) compared to patients with lower SAT.

### 3.6 Baseline total adipose tissue, visceral-to-subcutaneous fat tissue ratio , and overall survival and progression-free survival

**Figure 6:**
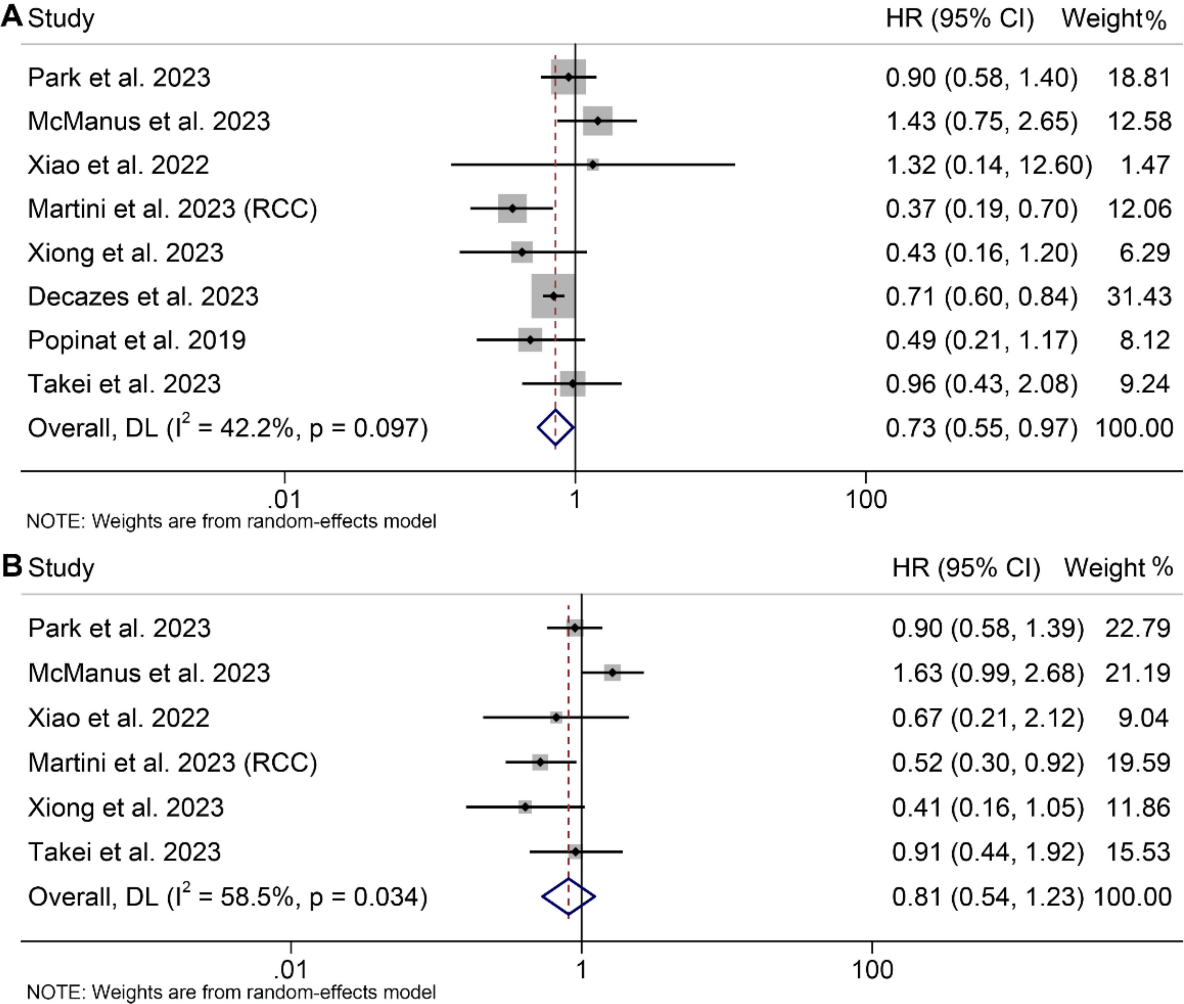
Forest plots of the relationship between total adipose tissue and overall survival **(A)** and progression-free survival **(B)**. HR, hazard ratio; CI, confidence interval; DL, DerSimonian and Laird.

Eight studies, involving 1297 patients, and five studies, involving 506 patients, investigated the predictive roles of TAT and VSR on the prognosis of cancer patients, respectively. The results indicated that cancer patients with elevated TAT exhibited significantly longer OS (I^2^ = 42.4%, *p* = 0.097, HR: 0.73, 95% CI: 0.55–0.97, *p* = 0.028, depicted in **Figure 6A**), while those with high VSR demonstrated a shorter OS (I^2^ = 0, *p* = 0.532, HR: 1.43, 95% CI: 1.09–1.87, *p* = 0.010, **Supplementary Figure S5A**). However, we found no correlation between the TAT (I^2^ = 58.5%, *p* = 0.034, HR: 0.81, 95% CI: 0.54–1.23, *p* = 0.332, **Figure 6B**) and VSR (I^2^ = 0, *p* = 0.757, HR: 1.20, 95% CI: 0.95–1.51, *p* = 0.131, **Supplementary Figure S5B**) and PFS in cancer patients treated with ICIs. The results of the subgroup analyses are detailed in **Table 2**.

Begg’s test, and Egger’s test did not reveal significant publication bias in OS (TAT, Egger’s test: *p* = 0.970, Begg’s test: *p* = 0.536; VSR, Egger’s test: *p* = 0.636, Begg’s test: *p* = 0.462) and PFS (TAT, Egger’s test: *p* = 0.368, Begg’s test: *p* = 0.452; VSR, Egger’s test: *p* = 0.785, Begg’s test: *p* = 1.000). The sensitivity analysis, in which each study was excluded one at a time, demonstrated that the pooled HRs for OS and PFS remained stable and robust (**Supplementary Figures S6A–D**).

### 3.7 Baseline intramuscular adipose tissue and prognosis and response

In assessing the correlation between IMAT and prognosis as well as response among cancer patients undergoing ICIs, an analysis was performed on seven cohorts comprising 511 individuals. Utilizing a random-effects model, it revealed no statistically significant differences in OS (I^2^ = 68.2%, *p* = 0.004, HR: 0.95, 95% CI: 0.62–1.46, *p* = 0.827, **Supplementary Figure S7A**), PFS (I^2^ = 68.6%, *p* = 0.007, HR: 0.91, 95% CI: 0.60–1.38, *p* = 0.654, **Supplementary Figure S7B**), and DCR (I^2^ = 63.5%, *p* = 0.042, HR: 1.03, 95% CI: 0.37–2.68, *p* = 0.956, **Supplementary Figure S7C**) between patients with high and low IMAT levels. The results of the subgroup analyses are detailed in **Table 2**.

Examination for potential publication bias via Begg’s test and Egger’s test did not reveal significant concerns regarding OS (Egger’s test: *p* = 0.724, Begg’s test: *p* = 0.548), PFS (Egger’s test: *p* = 0.880, Begg’s test: *p* = 1.000), and DCR (Egger’s test: *p* = 0.473, Begg’s test: *p* = 0.734). Our sensitivity analysis, involving the systematic exclusion of each study in sequence, consistently indicated the sustained stability and robustness of the pooled HRs for OS, PFS, and DCR (**Supplementary Figures S8A–C**).

## 4 Discussion

In the current study, we discovered that patients with high VAT and SAT had significantly longer survival and a higher therapeutic response. In contrast, TAT, VSR, and IMAT are not better predictors of prognosis in cancer patients treated with immunotherapy.

There has been a growing focus on the physiological implications of obesity and its influence on cancer therapy outcomes. The prevalence of obesity in the United States, currently affecting around 40% of the population, reflects a global trend where obesity rates have nearly tripled since 1976 (52). The Centers for Disease Control and Prevention have identified obesity as a heightened risk factor for 13 different cancer types (53, 54). Currently, adipose tissue has been identified as a lymphoid tissue, a concept particularly pertinent in understanding its implications in cancer and immunotherapy (55). The accumulation of white fat within the body has been proposed as a primary contributor to obesity and its associated complications (56). It is noteworthy that white adipose tissue serves as a reservoir for memory T cells, contributing to immune regulation (57). Obesity alters the composition and abundance of immune cell populations, including neutrophils, macrophages, B cells, and T cells (58). Immunotherapy directly supports the body's immune response against cancer (59). Considering the interaction between adipose tissue and the immune system, along with the reliance of immunotherapy agents on host immunity, it is plausible that the quantity and types of fat influence clinical responses to immunotherapy treatment. However, the specific impact of different fat depots on clinical outcomes in cancer patients undergoing immunotherapy has not been systematically explored in the literature to our knowledge.

Emerging data indicate a conflicting role of obesity and adipose tissue, along with their associated adipokines, as potential regulators in cancer-related mechanisms, exhibiting both tumor-promoting and tumor-suppressive effects. While adipose tissue is traditionally viewed as an endocrine organ, its role as a crucial modulator of the immune system is gaining recognition (60–62). Fat contributes to inflammation through the release of adipokines, cytokine-like molecules that sustain a state of chronic, low-grade inflammation (60). Among these adipokines, leptin stands out as a well-studied mediator linking metabolism and immune function, enhancing the population and function of regulatory T cells (63). Leptin has also been implicated in promoting immune evasion in lung cancer by upregulating proinflammatory cytokines (64). Obesity contributes to heightened PD-1 expression and increased release of PD-1 protein from T cells, along with elevated secretion of adiponectin and leptin from adipose tissue (65–67). These factors collectively contribute to enhanced T cell exhaustion and dysfunction, thereby facilitating tumor growth and progression (67). Thus, this suggests a potential mechanism for obesity-driven tumor immune escape, which can potentially be reversed through ICI therapy, leading to heightened effector T-cell responses. Collectively, obesity-induced chronic inflammation may contribute to tumorigenesis, yet increased adipose tissue may paradoxically enhance the host immune response to ICIs.

Adipose tissue is distributed in the visceral fat area and the subcutaneous fat area, which have different structural and functional characteristics. Our research findings revealed a significant association between VAT, SAT, and both survival and response. Thus, through the establishment of evidence-based data, it is crucial to consider the favorable impact of VAT on the efficacy of ICI therapy in clinical practice. Further research is warranted to explore whether controlling VAT and SAT improves the effectiveness of ICI therapy.

Certain limitations should be acknowledged in this meta-analysis. Firstly, it is worth mentioning that all investigations incorporated in this analysis were retrospective cohort studies, potentially constraining their statistical robustness. Additionally, due to the limited number of included studies, we were unable to perform subgroup analyses for specific cancer types and ICIs. Finally, the cut-off values for the same diagnostic metric differed among investigations. Hence, to attain more reliable conclusions, there is an urgent requirement for a worldwide, multicenter investigation to explore the impact of body fat tissue on outcomes in cancer patients undergoing ICIs.

## 5 Conclusion

The predictive effect of VAT and SAT on outcomes in ICI-treated cancer patients is highlighted by this analysis. This finding favors considering the VAT and SAT levels when determining the prognosis for this patient population.

## Data Availability

The original contributions presented in the study are included in the article/**Supplementary Material**. Further inquiries can be directed to the corresponding authors.

## References

[1] Hanahan D Weinberg RA. Hallmarks of cancer: the next generation. Cell. (2011) 144:646–74. doi: 10.1016/j.cell.2011.02.013 21376230

[2] Yuan Q Deng D Pan C Ren J Wei T Wu Z. Integration of transcriptomics, proteomics, and metabolomics data to reveal HER2-associated metabolic heterogeneity in gastric cancer with response to immunotherapy and neoadjuvant chemotherapy. Front Immunol. (2022) 13:951137. doi: 10.3389/fimmu.2022.951137 35990657 PMC9389544

[3] Pardoll DM. The blockade of immune checkpoints in cancer immunotherapy. Nat Rev Cancer. (2012) 12:252–64. doi: 10.1038/nrc3239 PMC4856023 22437870

[4] Larkin J Chiarion-Sileni V Gonzalez R Grob JJ Rutkowski P Lao CD. Five-year survival with combined nivolumab and ipilimumab in advanced melanoma. N Engl J Med. (2019) 381:1535–46. doi: 10.1056/NEJMoa1910836 31562797

[5] Ansell SM Lesokhin AM Borrello I Halwani A Scott EC Gutierrez M. PD-1 blockade with nivolumab in relapsed or refractory Hodgkin's lymphoma. N Engl J Med. (2015) 372:311–9. doi: 10.1056/NEJMoa1411087 PMC4348009 25482239

[6] Larkin J Chiarion-Sileni V Gonzalez R Grob JJ Cowey CL Lao CD. Combined nivolumab and ipilimumab or monotherapy in untreated melanoma. N Engl J Med. (2015) 373:23–34. doi: 10.1056/NEJMoa1504030 26027431 PMC5698905

[7] Cheng W Fu D Xu F Zhang Z. Unwrapping the genomic characteristics of urothelial bladder cancer and successes with immune checkpoint blockade therapy. Oncogenesis. (2018) 7:2. doi: 10.1038/s41389-017-0013-7 29358573 PMC5833720

[8] Ribas A Wolchok JD. Cancer immunotherapy using checkpoint blockade. Science. (2018) 359:1350–5. doi: 10.1126/science.aar4060 PMC7391259 29567705

[9] O'Donnell JS Teng MWL Smyth MJ. Cancer immunoediting and resistance to T cell-based immunotherapy. Nat Rev Clin Oncol. (2019) 16:151–67. doi: 10.1038/s41571-018-0142-8 30523282

[10] Dolladille C Ederhy S Sassier M Cautela J Thuny F Cohen AA. Immune checkpoint inhibitor rechallenge after immune-related adverse events in patients with cancer. JAMA Oncol. (2020) 6:865–71. doi: 10.1001/jamaoncol.2020.0726 PMC7163782 32297899

[11] Zhang L Feng J Kuang T Chai D Qiu Z Deng W. Blood biomarkers predict outcomes in patients with hepatocellular carcinoma treated with immune checkpoint Inhibitors: A pooled analysis of 44 retrospective sudies. Int Immunopharmacol. (2023) 118:110019. doi: 10.1016/j.intimp.2023.110019 36933492

[12] Zhao J Li D Xie S Deng X Wen X Li J. Nomogram for predicting prognosis of patients with metastatic melanoma after immunotherapy: A Chinese population-based analysis. Front Immunol. (2022) 13:1083840. doi: 10.3389/fimmu.2022.1083840 36618343 PMC9815596

[13] Ren J Wang A Liu J Yuan Q. Identification and validation of a novel redox-related lncRNA prognostic signature in lung adenocarcinoma. Bioengineered. (2021) 12:4331–48. doi: 10.1080/21655979.2021.1951522 PMC8806475 34338158

[14] Havel JJ Chowell D Chan TA. The evolving landscape of biomarkers for checkpoint inhibitor immunotherapy. Nat Rev Cancer. (2019) 19:133–50. doi: 10.1038/s41568-019-0116-x PMC6705396 30755690

[15] Zhou KI Peterson B Serritella A Thomas J Reizine N Moya S. Spatial and Temporal Heterogeneity of PD-L1 Expression and Tumor Mutational Burden in Gastroesophageal Adenocarcinoma at Baseline Diagnosis and after Chemotherapy. Clin Cancer Res. (2020) 26:6453–63. doi: 10.1158/1078-0432.CCR-20-2085 PMC7744325 32820017

[16] Jørgensen JT. Companion and complementary diagnostics: clinical and regulatory perspectives. Trends Cancer. (2016) 2:706–12. doi: 10.1016/j.trecan.2016.10.013 28741518

[17] Schalper KA Carleton M Zhou M Chen T Feng Y Huang SP. Elevated serum interleukin-8 is associated with enhanced intratumor neutrophils and reduced clinical benefit of immune-checkpoint inhibitors. Nat Med. (2020) 26:688–92. doi: 10.1038/s41591-020-0856-x PMC8127102 32405062

[18] Wang Y Wang J Liu J Zhu H. Immune-related diagnostic markers for benign prostatic hyperplasia and their potential as drug targets. Front Immunol. (2024) 15:1516362. doi: 10.3389/fimmu.2024.1516362 39703506 PMC11655502

[19] Pang ZQ Wang JS Wang JF Wang YX Ji B Xu YD. JAM3: A prognostic biomarker for bladder cancer via epithelial-mesenchymal transition regulation. Biomol Biomed. (2024) 24:897–911. doi: 10.17305/bb.2024.9979 38400838 PMC11293228

[20] Xiao J Mazurak VC Olobatuyi TA Caan BJ Prado CM. Visceral adiposity and cancer survival: a review of imaging studies. Eur J Cancer Care (Engl). (2018) 27:e12611. doi: 10.1111/ecc.2018.27.issue-2 27921375

[21] Hu JB Ravichandran S Rushing C Beasley GM Hanks BA Jung SH. But not sarcopenia, is associated with pembrolizumab-related toxicity in patients with advanced melanoma. Anticancer Res. (2020) 40:5245–54. doi: 10.21873/anticanres.14528 32878813

[22] Collet L Delrieu L Bouhamama A Crochet H Swalduz A Nerot A. Association between body mass index and survival outcome in metastatic cancer patients treated by immunotherapy: analysis of a french retrospective cohort. Cancers (Basel). (2021) 13(9):2200. doi: 10.3390/cancers13092200 34063692 PMC8124396

[23] Crombé A Kind M Toulmonde M Italiano A Cousin S. Impact of CT-based body composition parameters at baseline, their early changes and response in metastatic cancer patients treated with immune checkpoint inhibitors. Eur J Radiol. (2020) 133:109340. doi: 10.1016/j.ejrad.2020.109340 33091834

[24] Martin L Birdsell L Macdonald N Reiman T Clandinin MT McCargar LJ. Cancer cachexia in the age of obesity: skeletal muscle depletion is a powerful prognostic factor, independent of body mass index. J Clin Oncol. (2013) 31:1539–47. doi: 10.1200/JCO.2012.45.2722 23530101

[25] Deng J Huang Y Yu K Luo H Zhou D Li D. Changes in the gut microbiome of patients with esophageal cancer: A systematic review and meta-analysis based on 16S gene sequencing technology. Microb Pathog. (2024) 193:106784. doi: 10.1016/j.micpath.2024.106784 38971508

[26] Lilong Z Kuang T Li M Li X Hu P Deng W. Sarcopenia affects the clinical efficacy of immune checkpoint inhibitors in patients with gastrointestinal cancers. Clin Nutr. (2024) 43:31–41. doi: 10.1016/j.clnu.2023.11.009 38000193

[27] Egger M Davey Smith G Schneider M Minder C. Bias in meta-analysis detected by a simple, graphical test. Bmj. (1997) 315:629–34. doi: 10.1136/bmj.315.7109.629 PMC2127453 9310563

[28] Begg CB Mazumdar M. Operating characteristics of a rank correlation test for publication bias. Biometrics. (1994) 50:1088–101. doi: 10.2307/2533446 7786990

[29] Popinat G Cousse S Goldfarb L Becker S Gardin I Salaün M. Sub-cutaneous Fat Mass measured on multislice computed tomography of pretreatment PET/CT is a prognostic factor of stage IV non-small cell lung cancer treated by nivolumab. Oncoimmunology. (2019) 8:e1580128. doi: 10.1080/2162402X.2019.1580128 31069139 PMC6492978

[30] Martini DJ Kline MR Liu Y Shabto JM Williams MA Khan AI. Adiposity may predict survival in patients with advanced stage cancer treated with immunotherapy in phase 1 clinical trials. Cancer. (2020) 126:575–82. doi: 10.1002/cncr.v126.3 31648379

[31] Minami S Ihara S Tanaka T Komuta K. Sarcopenia and visceral adiposity did not affect efficacy of immune-checkpoint inhibitor monotherapy for pretreated patients with advanced non-small cell lung cancer. World J Oncol. (2020) 11:9–22. doi: 10.14740/wjon1225 32095185 PMC7011908

[32] Baldessari C Pecchi A Marcheselli R Guaitoli G Bonacini R Valoriani F. Body composition and inflammation impact in non-small-cell lung cancer patients treated by first-line immunotherapy. Immunotherapy. (2021) 13:1501–19. doi: 10.2217/imt-2021-0038 34670403

[33] Faron A Opheys NS Nowak S Sprinkart AM Isaak A Theis M. Deep learning-based body composition analysis predicts outcome in melanoma patients treated with immune checkpoint inhibitors. Diagn (Basel). (2021) 11(12):2314. doi: 10.3390/diagnostics11122314 PMC8700660 34943551

[34] Martini DJ Olsen TA Goyal S Liu Y Evans ST Magod B. Body composition variables as radiographic biomarkers of clinical outcomes in metastatic renal cell carcinoma patients receiving immune checkpoint inhibitors. Front Oncol. (2021) 11:707050. doi: 10.3389/fonc.2021.707050 34307176 PMC8299332

[35] Martini DJ Shabto JM Goyal S Liu Y Olsen TA Evans ST. Body composition as an independent predictive and prognostic biomarker in advanced urothelial carcinoma patients treated with immune checkpoint inhibitors. Oncologist. (2021) 26:1017–25. doi: 10.1002/onco.13922 PMC8649001 34342095

[36] Aslan V Kılıç ACK Sütcüoğlu O Eraslan E Bayrak A Öksüzoğlu B. Cachexia index in predicting outcomes among patients receiving immune checkpoint inhibitor treatment for metastatic renal cell carcinoma. Urol Oncol. (2022) 40:494.e1–.e10. doi: 10.1016/j.urolonc.2022.07.018 36137881

[37] Bolte FJ McTavish S Wakefield N Shantzer L Hubbard C Krishnaraj A. Association of sarcopenia with survival in advanced NSCLC patients receiving concurrent immunotherapy and chemotherapy. Front Oncol. (2022) 12:986236. doi: 10.3389/fonc.2022.986236 36212442 PMC9539742

[38] Lee JH Hyung S Lee J Choi SH. Visceral adiposity and systemic inflammation in the obesity paradox in patients with unresectable or metastatic melanoma undergoing immune checkpoint inhibitor therapy: a retrospective cohort study. J Immunother Cancer. (2022) 10(8):e005226. doi: 10.1136/jitc-2022-005226 36002189 PMC9413167

[39] Takenaka Y Takemoto N Otsuka T Nishio M Tanida M Fujii T. Predictive significance of body composition indices in patients with head and neck squamous cell carcinoma treated with nivolumab: A multicenter retrospective study. Oral Oncol. (2022) 132:106018. doi: 10.1016/j.oraloncology.2022.106018 35835055

[40] Xiao LS Li RN Cui H Hong C Huang CY Li QM. Use of computed tomography-derived body composition to determine the prognosis of patients with primary liver cancer treated with immune checkpoint inhibitors: a retrospective cohort study. BMC Cancer. (2022) 22:737. doi: 10.1186/s12885-022-09823-7 35794525 PMC9258103

[41] Decazes P Ammari S Belkouchi Y Mottay L Lawrance L de Prévia A. Synergic prognostic value of 3D CT scan subcutaneous fat and muscle masses for immunotherapy-treated cancer. J Immunother Cancer. (2023) 11(9):e007315. doi: 10.1136/jitc-2023-007315 37678919 PMC10496660

[42] Khan A Welman CJ Abed A O'Hanlon S Redfern A Azim S. Association of computed tomography measures of muscle and adipose tissue and progressive changes throughout treatment with clinical endpoints in patients with advanced lung cancer treated with immune checkpoint inhibitors. Cancers (Basel). (2023) 15(5):1382. doi: 10.3390/cancers15051382 36900175 PMC10000131

[43] Makrakis D Rounis K Tsigkas AP Georgiou A Galanakis N Tsakonas G. Effect of body tissue composition on the outcome of patients with metastatic non-small cell lung cancer treated with PD-1/PD-L1 inhibitors. PloS One. (2023) 18:e0277708. doi: 1371/journal.pone.0277708 36763597 10.1371/journal.pone.0277708 PMC9916610

[44] McManus HD Zhang D Schwartz FR Wu Y Infield J Ho E. Relationship between pretreatment body composition and clinical outcomes in patients with metastatic renal cell carcinoma receiving first-line ipilimumab plus nivolumab. Clin Genitourin Cancer. (2023) 21:e429–e37.e2. doi: 10.1016/j.clgc.2023.05.006 37271698

[45] Park JE Jo J Youk J Kim M Yoon SH Keam B. Prognostic utility of body composition parameters based on computed tomography analysis of advanced non-small cell lung cancer treated with immune checkpoint inhibitors. Insights Imaging. (2023) 14:182. doi: 10.1186/s13244-023-01532-4 37880430 PMC10600077

[46] Takei K Kijima T Okubo N Kurashina R Kokubun H Uematsu T. Association between immune checkpoint inhibitor treatment outcomes and body composition factors in metastatic renal cell carcinoma patients. Cancers (Basel). (2023) 15(23):5591. doi: 10.3390/cancers15235591 38067295 PMC10705346

[47] Tanaka T Miwa K Shimotsuura Y Nagasu S Shigyou H Hirota K. High intramuscular adipose tissue content was a favorable prognostic factor in patients with advanced gastric cancer treated with nivolumab monotherapy. J Gastroenterol Hepatol. (2023) 38:1760–7. doi: 10.1111/jgh.v38.10 37225648

[48] Uojima H Chuma M Hidaka H Tsuda T Kobayashi S Hattori N. Impact of body composition for patients with hepatocellular carcinoma who received atezolizumab plus bevacizumab therapy. Eur J Gastroenterol Hepatol. (2023) 35:865–73. doi: 10.1097/MEG.0000000000002581 37395239

[49] Wang J Dong P Qu Y Xu W Zhou Z Ning K. Association of computed tomography-based body composition with survival in metastatic renal cancer patient received immunotherapy: a multicenter, retrospective study. Eur Radiol. (2023) 33:3232–42. doi: 10.1007/s00330-022-09345-7 36538073

[50] Xiong B Fu B Wu Y Gao F Hou C. Body composition predicts prognosis of hepatocellular carcinoma patients undergoing immune checkpoint inhibitors. J Cancer Res Clin Oncol. (2023) 149:11607–17. doi: 10.1007/s00432-023-05051-z PMC11797616 37400572

[51] Zhang J Yang X Yang X Xu J Wang Y Wang Y. Prognostic value of SAT volume and density for predicting the outcome of patients with unresectable HCC treated with lenvatinib plus anti-PD-1 antibodies. Am J Cancer Res. (2023) 13:912–21. PMC10077037 37034208

[52] Colotta F Allavena P Sica A Garlanda C Mantovani A. Cancer-related inflammation, the seventh hallmark of cancer: links to genetic instability. Carcinogenesis. (2009) 30:1073–81. doi: 10.1093/carcin/bgp127 19468060

[53] Budui SL Rossi AP Zamboni M. The pathogenetic bases of sarcopenia. Clin cases Miner Bone Metab. (2015) 12:22–6. doi: 10.11138/ccmbm/2015.12.1.022 PMC4469221 26136791

[54] Wang JF Wang JS Liu Y Ji B Ding BC Wang YX. Knockdown of integrin β1 inhibits proliferation and promotes apoptosis in bladder cancer cells. Biofactors. (2025) 51:e2150. doi: 10.1002/biof.v51.1 39644117

[55] Escobedo N Oliver G. The lymphatic vasculature: its role in adipose metabolism and obesity. Cell Metab. (2017) 26:598–609. doi: 10.1016/j.cmet.2017.07.020 28844882 PMC5629116

[56] Gesta S Tseng YH Kahn CR. Developmental origin of fat: tracking obesity to its source. Cell. (2007) 131:242–56. doi: 10.1016/j.cell.2007.10.004 17956727

[57] Han SJ Glatman Zaretsky A Andrade-Oliveira V Collins N Dzutsev A Shaik J. White adipose tissue is a reservoir for memory T cells and promotes protective memory responses to infection. Immunity. (2017) 47:1154–68.e6. doi: 10.1016/j.immuni.2017.11.009 29221731 PMC5773068

[58] Grant RW Dixit VD. Adipose tissue as an immunological organ. Obes (Silver Spring). (2015) 23:512–8. doi: 10.1002/oby.21003 PMC4340740 25612251

[59] Yang Y. Cancer immunotherapy: harnessing the immune system to battle cancer. J Clin Invest. (2015) 125:3335–7. doi: 10.1172/JCI83871 PMC4588312 26325031

[60] Francisco V Pino J Campos-Cabaleiro V Ruiz-Fernández C Mera A Gonzalez-Gay MA. Obesity, fat mass and immune system: role for leptin. Front Physiol. (2018) 9:640. doi: 10.3389/fphys.2018.00640 29910742 PMC5992476

[61] Wang Y Wang J He J Ji B Pang Z Wang J. Comprehensive analysis of PRPF19 immune infiltrates, DNA methylation, senescence-associated secretory phenotype and ceRNA network in bladder cancer. Front Immunol. (2023) 14:1289198. doi: 10.3389/fimmu.2023.1289198 38022515 PMC10657824

[62] Wang Y Zhu H Xu H Qiu Y Zhu Y Wang X. Senescence-related gene c-Myc affects bladder cancer cell senescence by interacting with HSP90B1 to regulate cisplatin sensitivity. Aging (Albany NY). (2023) 15:7408–23. doi: 10.18632/aging.204863 PMC10457043 37433010

[63] Matarese G Procaccini C De Rosa V Horvath TL La Cava A. Regulatory T cells in obesity: the leptin connection. Trends Mol Med. (2010) 16:247–56. doi: 10.1016/j.molmed.2010.04.002 20493774

[64] Shen Y Wang Q Zhao Q Zhou J. Leptin promotes the immune escape of lung cancer by inducing proinflammatory cytokines and resistance to apoptosis. Mol Med Rep. (2009) 2:295–9. doi: 10.3892/mmr_00000099 21475828

[65] Finelli C. Obesity and immunotherapy: the surprisingly positive association! Immunotherapy. (2020) 12:541–4. doi: 10.2217/imt-2019-0143 32345093

[66] Zhang X Liu Y Shao H Zheng X. Obesity paradox in lung cancer prognosis: evolving biological insights and clinical implications. J Thorac Oncol. (2017) 12:1478–88. doi: 10.1016/j.jtho.2017.07.022 28757418

[67] Wang Z Aguilar EG Luna JI Dunai C Khuat LT Le CT. Paradoxical effects of obesity on T cell function during tumor progression and PD-1 checkpoint blockade. Nat Med. (2019) 25:141–51. doi: 10.1038/s41591-018-0221-5 PMC6324991 30420753

