# Supplementary material for: Prognostic value of body adipose tissue parameters in cancer patients treated with immune checkpoint inhibitors
Source: Front Immunol. 2025 Feb 12;16:1557726. doi: 10.3389/fimmu.2025.1557726 (PMC11861556; doi:10.3389/fimmu.2025.1557726)
Supplement: Supplementary file 1 [file DataSheet1.docx]

Supplementary material 1. Detailed article search strategy

(((((((((((((((((((((((((((((((((((((((((((((((((Camrelizumab) OR (Sintilimab)) OR (Tislelizumab)) OR (Toripalimab)) OR (Envafolimab)) OR (Immune Checkpoint Inhibitors)) OR (Checkpoint Inhibitors, Immune)) OR (Immune Checkpoint Inhibitor)) OR (Checkpoint Inhibitor, Immune)) OR (Immune Checkpoint Blockers)) OR (Checkpoint Blockers, Immune)) OR (Immune Checkpoint Blockade)) OR (Checkpoint Blockade, Immune)) OR (Immune Checkpoint Inhibition)) OR (Checkpoint Inhibition, Immune)) OR (PD-L1 Inhibitors)) OR (PD L1 Inhibitors)) OR (PD-L1 Inhibitor)) OR (PD L1 Inhibitor)) OR (Programmed Death-Ligand 1 Inhibitors)) OR (Programmed Death Ligand 1 Inhibitors)) OR (PD-1-PD-L1 Blockade)) OR (Blockade, PD-1-PD-L1)) OR (PD 1 PD L1 Blockade)) OR (CTLA-4 Inhibitors)) OR (CTLA 4 Inhibitors)) OR (CTLA-4 Inhibitor)) OR (CTLA 4 Inhibitor)) OR (Cytotoxic T-Lymphocyte-Associated Protein 4 Inhibitors)) OR (Cytotoxic T Lymphocyte Associated Protein 4 Inhibitors)) OR (Cytotoxic T-Lymphocyte-Associated Protein 4 Inhibitor)) OR (Cytotoxic T Lymphocyte Associated Protein 4 Inhibitor)) OR (PD-1 Inhibitors)) OR (PD-1 Inhibitor)) OR (PD 1 Inhibitors)) OR (Inhibitor, PD-1)) OR (PD 1 Inhibitor)) OR (Programmed Cell Death Protein 1 Inhibitor)) OR (Programmed Cell Death Protein 1 Inhibitors)) OR (Pembrolizumab)) OR (Nivolumab)) OR (Atezolizumab)) OR (Ipilimumab)) OR (Avelumab)) OR (Tremelimumab)) OR (Durvalumab)) OR (Cemiplimab)) OR (Immune Checkpoint Inhibitors[MeSH Terms]))) AND (((Subcutaneous Adipose Index) OR (Subcutaneous Adipose Area) OR (Subcutaneous Adipose Tissue) OR (Subcutaneous Adipose Mass) OR (Subcutaneous Fat Index) OR (Subcutaneous Fat Area) OR (Subcutaneous Fat Tissue) OR (Subcutaneous Fat Mass) OR (Intramuscular Adipose content) OR (Intramuscular Adipose Index) OR (Intramuscular Adipose Area) OR (Intramuscular Adipose Tissue) OR (Intramuscular Adipose Mass) OR (Intramuscular Fat Index) OR(Intramuscular Fat Area) OR (Intramuscular Fat Tissue) OR (Intramuscular Fat Mass) OR (Intramuscular Fat Content) OR (Visceral Adiposity) OR (Visceral Adipose Index) OR (Visceral Adipose Area) OR (Visceral Adipose Tissue) OR (Visceral Adipose Mass) OR (Visceral Fat Index) OR (Visceral Fat Tissue) OR (Visceral Fat Mass) OR (Visceral Fat Area) OR (Total Fat Index) OR (Total Fat Area) OR (Total Fat Tissue) OR (Total Fat Mass) OR (Total Adipose Index) OR (Total Adipose Tissue) OR (Total Adipose Area) OR (Total Adipose Mass)))


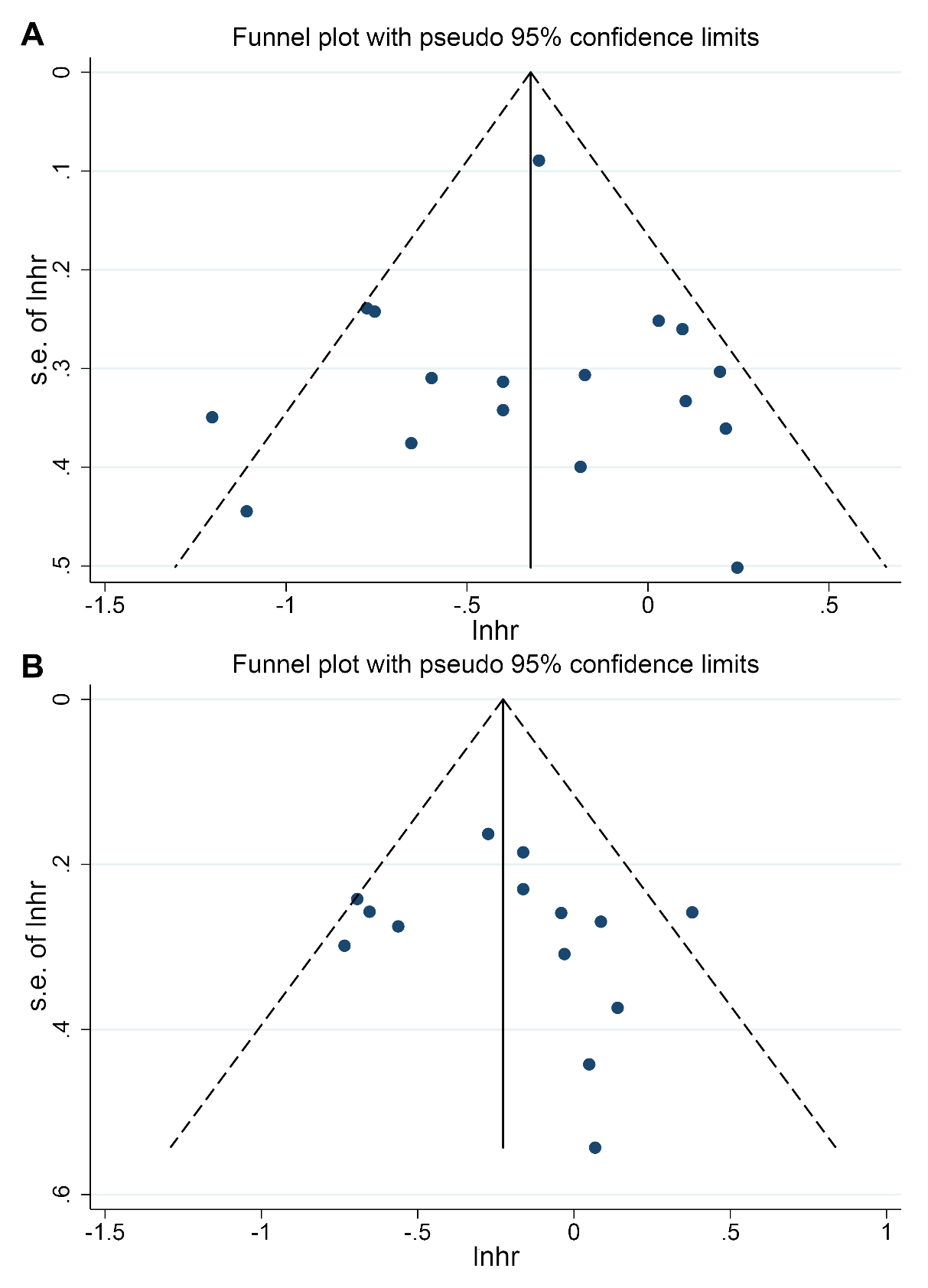


Figure S1. Funnel plots of the relationship between visceral adipose tissue and overall survival (A) and progression-free survival (B). HR, hazard ratio.


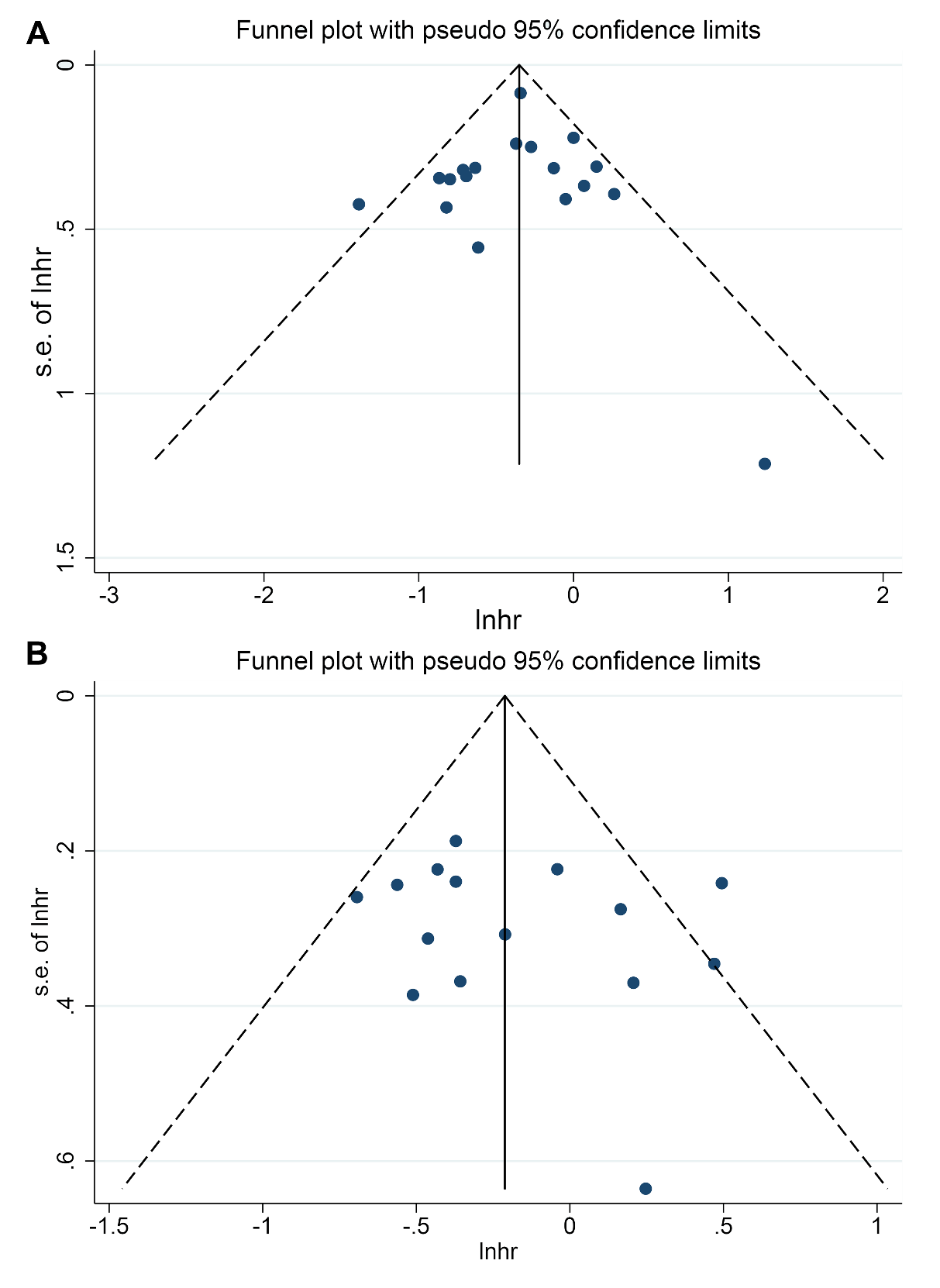


Figure S2. Funnel plots of the relationship between subcutaneous adipose tissue and overall survival (A) and progression-free survival (B). HR, hazard ratio.


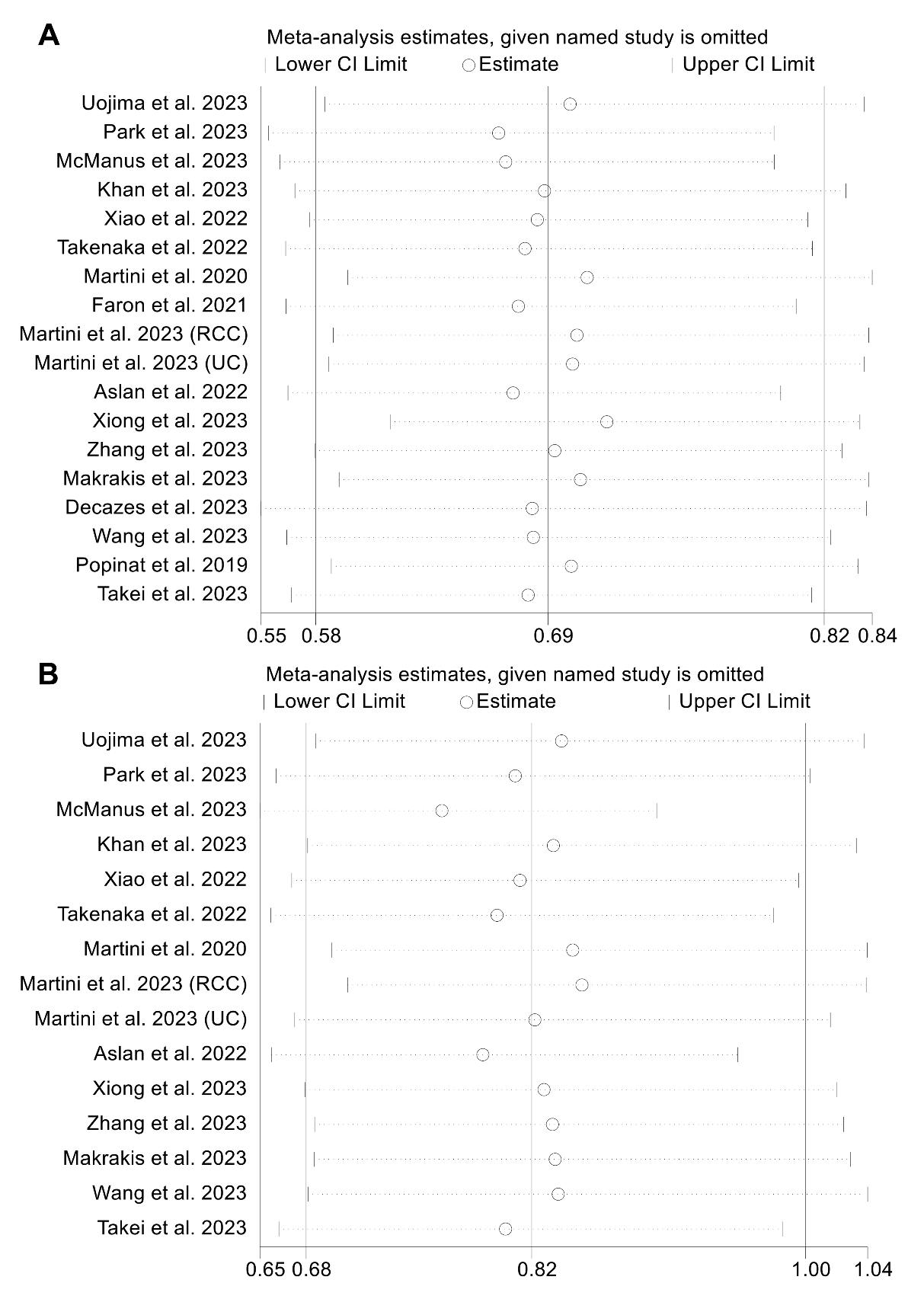


Figure S3. Sensitivity analysis of the association between subcutaneous adipose tissue and overall survival (A) and progression-free survival (B). HR, hazard ratio; CI, confidence interval.


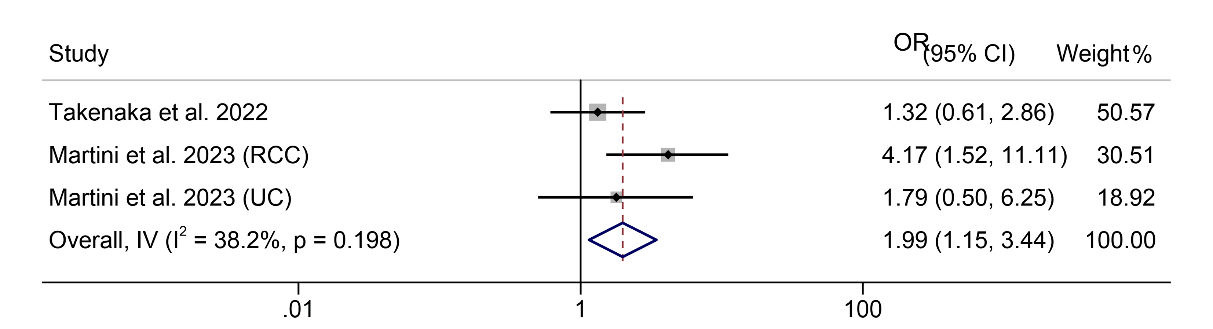


Figure S4. Forest plots of the relationship between subcutaneous adipose tissue and disease control rate. OR, odds ratio; CI, confidence interval.


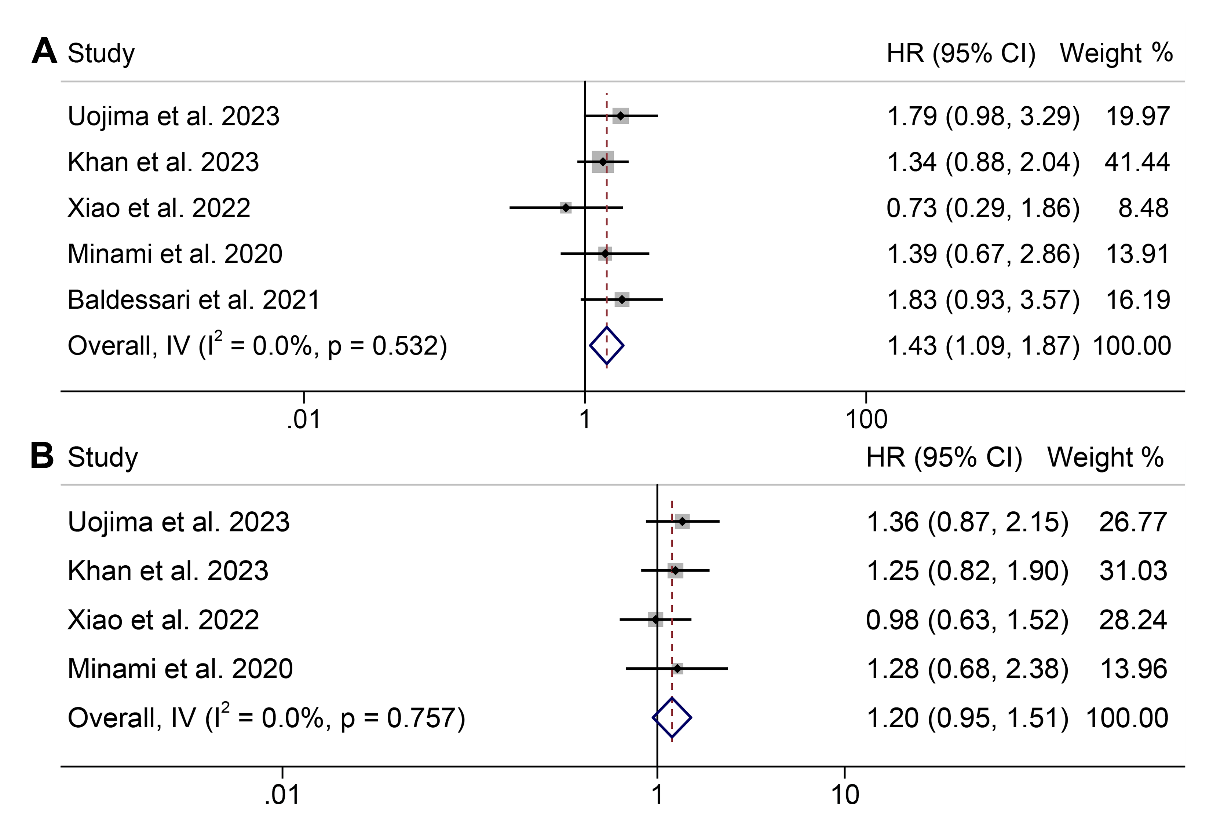


Figure S5. Forest plots of the relationship between visceral-to-subcutaneous fat tissue ratio and overall survival (A) and progression-free survival (B). HR, hazard ratio; CI, confidence interval.


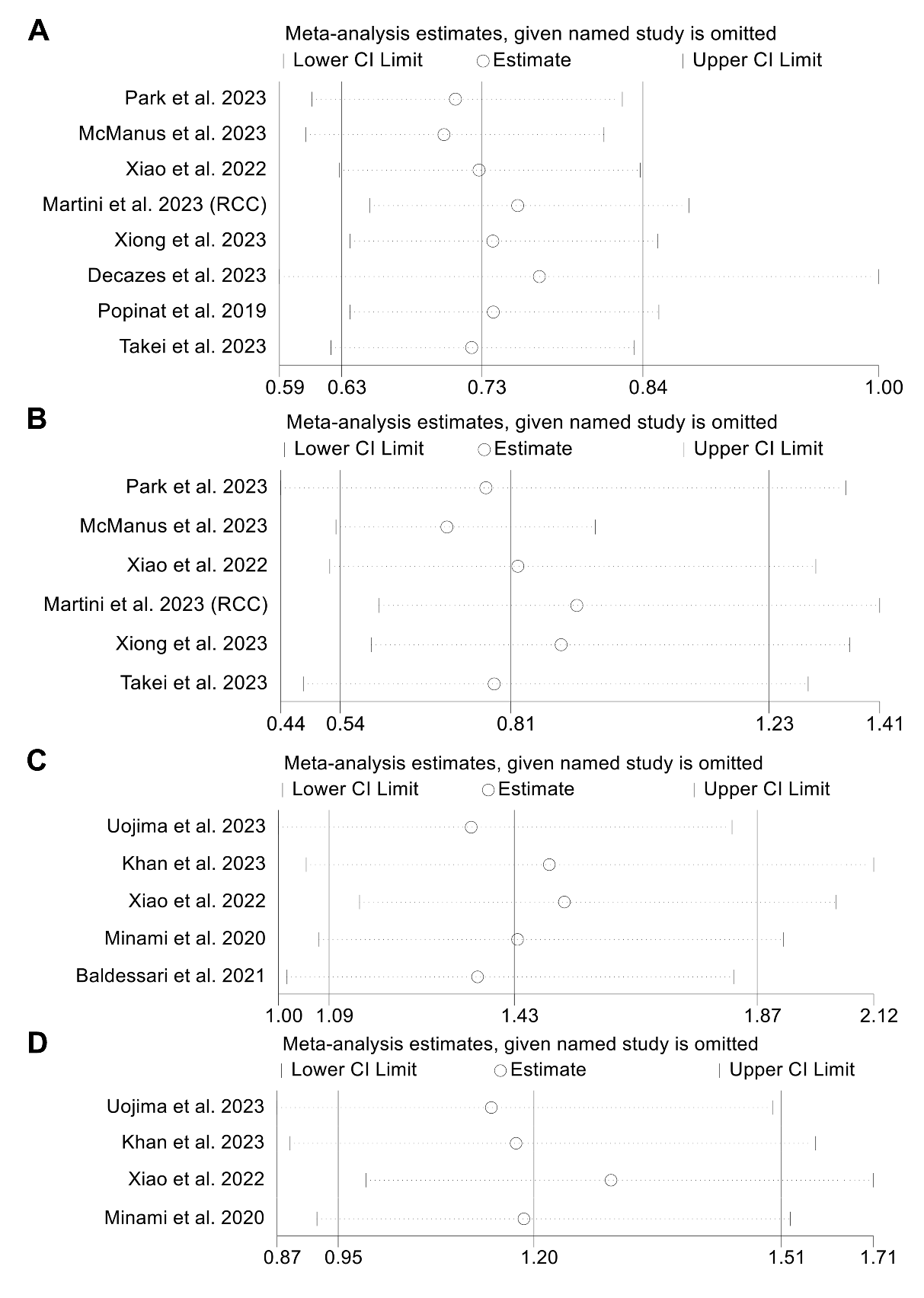


Figure S6. Sensitivity analysis of the association between total adipose tissue and overall survival (A) and progression-free survival (B). Sensitivity analysis of the association between visceral-to-subcutaneous fat tissue ratio and overall survival (C) and progression-free survival (D). HR, hazard ratio; CI, confidence interval.


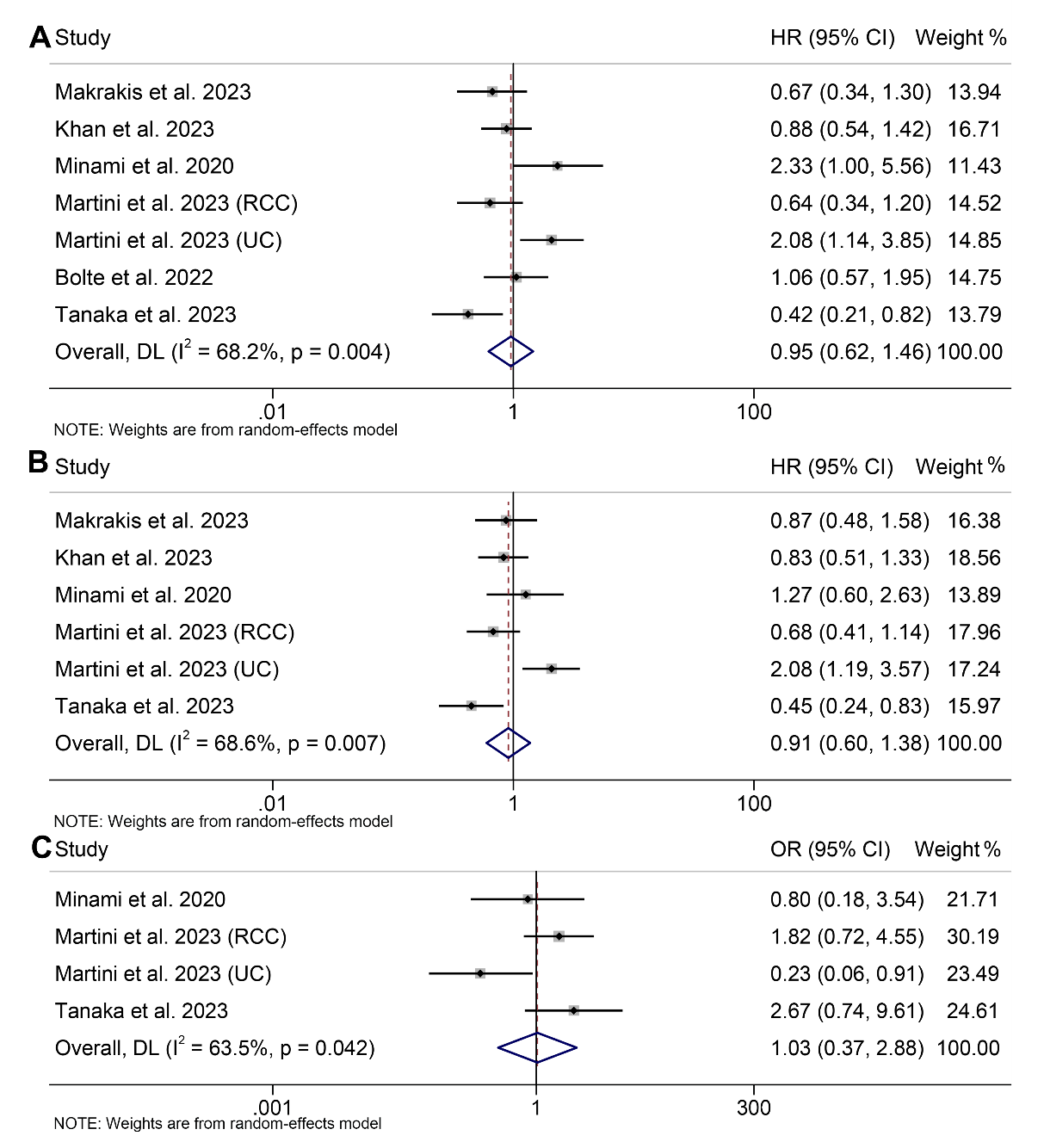


Figure S7. Forest plots of the relationship between intramuscular adipose tissue and overall survival (A), progression-free survival (B), and disease control rate (C). HR, hazard ratio; CI, confidence interval.


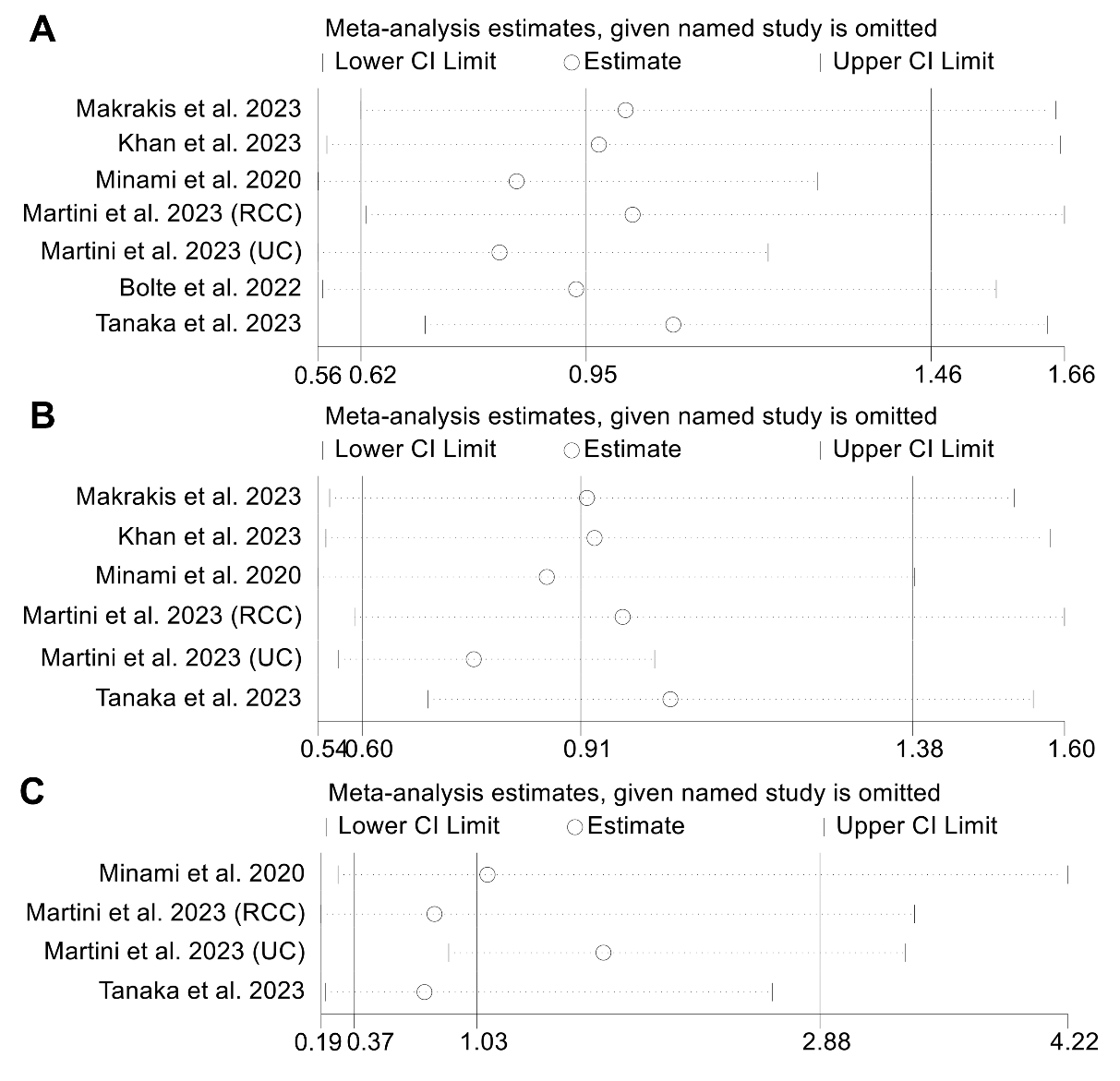


Figure S8. Sensitivity analysis of the association between total adipose tissue and overall survival (A), progression-free survival (B), and disease control rate (C). CI, confidence interval.
